# Surface-Modification Strategy to Produce Highly Anticorrosive Ti_3_C_2_T_x_ MXene-Based Polymer Composite Coatings: A Mini-Review

**DOI:** 10.3390/ma18030653

**Published:** 2025-02-01

**Authors:** Shufang Zhang, Guoqin Zhang, Liang Fang, Zhiheng Wang, Fang Wu, Gaobin Liu, Qirui Wang, Hongen Nian

**Affiliations:** 1 Key Laboratory of Green and High-End Utilization of Salt Lake Resources, Qinghai Institute of Salt Lake, Chinese Academy of Sciences, Xining 810008, China; roseymcn.cn2000@foxmail.com (S.Z.); qrwang@isl.ac.cn (Q.W.); 2 Chongqing Key Laboratory of Interface Physics in Energy Conversion, College of Physics, Chongqing University, Chongqing 400044, China; zgq@cqyu.edu.cn (G.Z.); 202027021033@cqu.edu.cn (Z.W.); wufang@cqu.edu.cn (F.W.); gbl@cqu.edu.cn (G.L.); 3 College of AI and BigData, Chongqing Polytechnic University of Electronic Technology, Chongqing 401331, China; 4 Aviation and Automobile School, Chongqing Youth Vocational & Technical College, Chongqing 400712, China; 5 Center of Modern Physics, Institute for Smart City of Chongqing University in Liyang, Liyang 213300, China

**Keywords:** MXene composites, Ti_3_C_2_T_x_, organic composite coatings, anticorrosion coatings, 2D materials, surface modification

## Abstract

MXenes are a group of novel two-dimensional (2D) materials with merits such as large specific surface area, abundant surface-functional groups, high chemical activity, excellent mechanical properties, high hydrophilicity, and good compatibility with various polymers. In recent years, many novel high-performance organic anticorrosion coatings using MXenes as nanofillers have been reported and have attracted widespread attention. As the first successfully prepared MXene material, Ti_3_C_2_T_x_ is the most extensively studied and typical member of the MXene family. Therefore, it is taken as the representative of its family, and the status of Ti_3_C_2_T_x_ MXene/epoxy resin (EP) and MXene/waterborne polyurethane (WPU) polymer anticorrosive composite coatings is reviewed. Firstly, the structure, characteristics, and main synthesis methods of MXenes are briefly introduced. Then, the latest progress of four surface-modification strategies to improve the dispersion, compatibility, stability, and anti-aggregation properties of MXenes, namely functionalization grafting, orientation regulation, heterostructure nanocomposite design, and stabilization and greening treatment, are analyzed and summarized. Finally, the current challenges and future opportunities regarding MXene-based corrosion-resistant organic composite coatings are discussed prospectively.

## 1. Introduction

Corrosion is a ubiquitous and costly issue for diverse industries [1,2,3,4,5,6,7,8]. The economic losses caused by corrosion are around USD 2.5 trillion annually for the whole world, as evaluated by the National Association for Corrosion Engineers (NACE) [1]. Additionally, as early as 2017, corrosion and related destruction were estimated to account for a loss of approximately RMB 2128 billion (≈USD 310 billion) in China, which is nearly 3.34% of the value of the gross domestic product (GDP) [2]. Effective corrosion control is important not only for economic savings but also for the safety, longevity, and sustainability of metal assets.

In order to minimize its adverse effects, various methods [9,10,11], such as protective coatings, surface treatments, and corrosion inhibitors, have been adopted to prevent corrosion. Among these, organic anticorrosion coatings [12,13,14,15,16] have the advantages of simplicity, low cost, high efficiency, and environmental friendliness and are widely used in fields such as construction, automobiles, aerospace, and ocean engineering. Organic anticorrosion coatings are usually based on epoxy resin (EP), polyurethane (PU), acrylic, or another polymer matrix [17,18,19,20] and commonly suffer from defects such as coating shrinkage, internal pores, cracks, and peeling caused by solvent evaporation. These defects introduce channels for corrosive media (such as water molecules and Cl^-^ ions) to diffuse into the surface of the metal substrate, weakening the protective effect of the coating. Therefore, there is an urgent need to develop protective coatings with as few internal defects (such as microholes) as possible.

The incorporation of fillers can enhance corrosion resistance by reducing defects such as pores in organic coatings, increasing coating density, and prolonging the diffusion path of corrosive substances [17,18,19,20]. Among various optional fillers, due to their large specific surface area, adjustable electronic structure, good flexibility, and excellent mechanical properties, two-dimension (2D) layered materials have great potential to improve the performance of protective coatings in blocking the penetration of corrosive ions. A variety of 2D materials such as graphene (Gr), graphene oxide (GO), hexagonal boron nitride (h-BN), molybdenum disulfide (MoS_2_), and layered double hydroxides (LDHs) have been successively attempted [21,22,23,24,25]. However, the cumbersome and costly preparation of Gr and h-BN and their inherent chemical inertness result in weak interactions with organic substrates and easy aggregation, leading to poor corrosion resistance.

In contrast, MXenes (2D transition metal carbides/nitrides) have abundant surface chemical terminal groups, which easily form bonds with other materials or undergo surface modification or functionalization to improve the corrosion resistance of organic coatings [26,27,28,29,30]. Therefore, MXenes have attracted widespread attention in recent years [31,32,33,34,35,36]. For example, in 2019, H. Yan et al. first introduced an MXene (Ti_3_C_2_T_x_) in the field of corrosion prevention [30]. Subsequently, research on new organic anticorrosion coatings based on MXenes has grown rapidly. However, actual MXenes may have many structural defects, and the characteristics of easy stacking, good conductivity, and proneness to oxidation are adverse to corrosion protection. Therefore, there is controversy about whether MXenes can be applied for corrosion protection or not. Thus, a few basic questions need to be answered, such as: if it can be applied, what are its advantages and disadvantages? How can it be used appropriately? Is there any special attention essential? Therefore, it is necessary to summarize and analyze the characteristics, performance, and development trends of MXene-based anticorrosion coatings.

Ti_3_C_2_T_x_ was the first successfully synthesized 2D material in the MXene class; as a result, it has been the most studied and has garnered constant interest from academia and industry. The MXene Ti_3_C_2_T_x_ possesses the highest electrical conductivity (up to 24,000 S·cm^−1^) and one of the highest stiffness values, with a Young’s modulus of ~334 GPa, among water-dispersible conductive 2D materials [3]. The negative surface charge of MXenes is conducive to their effective dispersion in aqueous and other polar solvents. There are many reviews on Ti_3_C_2_T_x_ polymer composites for catalysis, flexible electronics, and energy storage, but most of them are focused on the whole 2D family, not especially on MXenes or Ti_3_C_2_T_x_, resulting in insufficient depth and specificity in analysis and discussion. To the best of our knowledge, only one review particularly discussed Ti_3_C_2_T_x_/polymer composites for anticorrosion applications has been published, by I. Amin et al. in 2022 [3].

In order to report the current progress, especially after 2022, this mini-review first briefly introduces the structural characteristics and synthesis methods of MXene. Then, focusing on how to improve the dispersibility, compatibility, stability, and anti-agglomeration of MXene to enhance the corrosion resistance of organic composite coatings, four surface-modification methods on Ti_3_C_2_T_x_ MXene, namely functional grafting, orientation arrangement, heterostructure nanocomposite design, and stabilization and greening treatment, are discussed in detail. Finally, the current challenges and future development opportunities of MXene-based coatings in the field of anticorrosion are prospected.

The main reviews related to recent advances in the synthesis and application of corrosion-resistant MXene-based polymer composite materials published after 2022 are compared in the present review. The title, focus, and published year of these review papers are summarized in Table 1, which illustrates that the novelty and uniqueness of this review lie in summarizing and proposing four surface-modification strategies (functionalization grafting, orientation regulation, heterostructure hybrid, and stabilization and greening) to achieve high-performance anticorrosive Ti_3_C_2_T_x_ MXene-based polymer composite coating.

## 2. Brief Introduction of MXene and Overview of Ti_3_C_2_T_x_’s Anticorrosion

### 2.1. Structure and Main Preparation Methods of MXene

**Figure 1:**
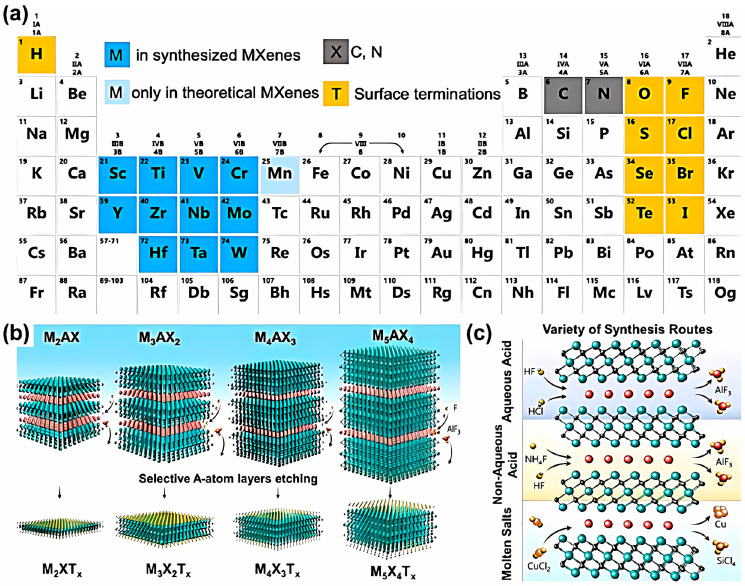
(**a**) Periodic table with color-coded elements that are adopted to construct MXenes. (**b**) Schematic representation of various MXenes (M2XTx, M3X2Tx, M4X3Tx, and M5X4Tx) obtained from the corresponding MAX phases (M2AX, M3AX2, M4AX3, and M5AX4) with the general formula Mn+1AXn (n = 1–4), where X is C and/or N; Tx is –H, =O, –F, and –OH in MXenes. (**c**) Schematic illustration of the three most employed synthesis routes for MXenes via aqueous acids (mixture of HCl/HF), nonaqueous acids (NH4F/HF), and molten salts [39]. Reprinted with permission from [39], copyright 2022, American Chemical Society.

MXene is a new category of 2D lamellar material discovered in 2011, derived from pre-transition metal carbides or nitrides of MAX phase [37,38,39,40], which can be written as M_n+1_AX_n_, where M denotes the pre-transition metal containing Sc, Ti, V, Cr, Zr, Nb, Mo, Hf, Ta, etc., A represents the third/fourth main group elements, such as Al, Ga, Ln, Tl, Si, Ge, Sn, Pb, etc., and X refers to C or N; n = 1, 2, or 3. MAX phase consists of layered ternary carbides and nitrides, with over 70 types reported so far. In the MAX crystal structure, the M layer is densely packed, with X atoms filling its octahedral position and A atoms alternating with M_n+1_X_n_ layers to form a layered structure. Removing the A element from MAX results in 2D MXene, commonly abbreviated as M_n+1_X_n_T_x_, where T_x_ indicates surface-functional groups such as =O, –OH, and –F left after etching, and n represents the number of atomic layers of the elements occurring in a sandwich-like layered morphology. In the periodic table, the elements used to build MXenes represented by different colors are shown in Figure 1a, and various MXenes (M_2_XT_x_, M_3_X_2_T_x_, M_4_X_3_T_x_, and M_5_X_4_T_x_) correspondingly obtained from MAX phases (M_2_AX, M_3_AX_2_, M_4_AX_3_, and M_5_AX_4_) with the general formula M_n+1_AX_n_ (n = 1–4) are demonstrated in Figure 1b [39].

Compared with other 2D materials, such as Gr, GO, BN, and MoS_2_, MXenes have the following main unique advantages [4]: (1) The intact structure gives it a better conductivity (1.1–2.4 MS/m) than GO and BN (ranges from 0.032 to 0.119 MS/m for GO). (2) The presence of various surface-functional groups, such as =O, –OH, and –F, gives it an intrinsic negative charge characteristic and hydrophilicity, which is beneficial to combine with the positively charged NMs, such as LDH, to prevent self-agglomeration. In contrast to GO with –COOH, the –OH on MXene causes it to be hydrophilic, being conducive to dispersing well in water-based solvents or polymers. (3) The formation of TiO_2_ on Ti-based MXene (Ti_3_C_2_T_x_, Ti_2_CT_x_, etc.) can further enhance the corrosion resistance of composite coatings because the formed nanoparticles can serve as filler in the coating. (4) Distinct from Gr or BN, MXene is a large family. Owing to the tunable compositions, multiple types of MXenes offer various kinds of functions and practical applications. Ti-, Cr-, V-, and Mo-based MXenes, like Ti_3_C_2_T_x_, Cr_3_C_2_T_x_, V_2_CT_x_, and Mo_2_CT_x_, and others, as well as the amorphous MXene and emerging high-entropy MXene (HE-MXene), provide the possibility for multi-functional coatings and present promising potential for various applications.

As for the preparation of MXenes, there are generally two strategies [41,42,43,44,45,46]: “bottom-up” or “top-down”. The former is based on templates and synthesized through chemical vapor deposition (CVD), while the latter is through peeling off MAX bulk crystals into several layers or single-layer sheets. At present, the more commonly used approach is “top-down”, which includes two main steps: etching and exfoliation. In the etching process, a strong corrosive agent is usually employed to selectively remove the A layer in the MAX material to obtain an “accordion” shaped material. Then, intercalation agents or mechanical vibrations such as ultrasound and ball milling are adopted for exfoliation (stripping) treatment, therefore achieving multi-, few-, or single-layer MXene. The three most employed methods to produce MXenes are though aqueous acid (HCl/HF mixture), nonaqueous acid (NH_4_F/HF), or molten salt. The corresponding synthetic routes are illustrated in Figure 1c [39].

Generally speaking, MXenes can be fabricated through diverse etching methods, but the MXenes obtained by various preparation approaches will possess different characteristics, such as different structures, surface chemical states, and physical or chemical properties. Therefore, it is necessary to select appropriate preparation and etching methods according to the special target application scenario and performance requirements of MXenes. For example, if high conductivity, large sheet size, wide interlayer spacing, and high mechanical performance are required, mild etching conditions (typically LiF + HCl) are recommended, but if MXenes with small sheet size and rich defects are wanted, HF etching may be the better choice.

Similar to Gr, owing to the large surface area, good chemical and mechanical stability, excellent conductivity, and high flexibility, extensive work and exploration on the application of MXene nanosheets have been carried out in various fields such as energy storage [47], electromagnetic shielding [48,49], adsorption, flexible devices, catalysis, superconductivity [45], and corrosion prevention.

So far, over 50 varieties of MXene have been reported to have been synthetized, and more than 100 have been explored theoretically [32]. Since the synthesis and properties of MXene and Ti_3_C_2_T_x_ have been discussed in numerous reviews elsewhere [50,51,52,53,54,55], they are not within the scope of this review.

### 2.2. Overview of Ti_3_C_2_T_x_ and Its Anticorrosion

As the first 2D MXene material obtained from the Ti_3_AlC_2_ MAX phase [37], Ti_3_C_2_T_x_ is the most studied one and has gained the greatest attention in the MXene class since its first synthesis in 2011. Ti_3_C_2_T_x_ is composed of three layers of Ti atoms and two layers of C atoms placed in the layers of Ti–C–Ti–C–Ti. The Tx component in the formula denotes the surface terminations (typically –OH, =O, –F, –Cl) existing on the outer planes of Ti. The negatively charged surface of Ti_3_C_2_T_x_ is beneficial to its excellent dispersion in aqueous and other polar solvents. The lamellar structure of Ti_3_C_2_T_x_, combined with its good solubility across a wide range of solvents, tunable surface functionality, excellent interface interaction, and stability with other organic/polymeric materials, makes it a potential material for anticorrosive coatings. In addition, the “top-down” synthesis route through wet chemical selective etching from its precursor, the Ti_3_AlC_2_ MAX phase, causes it to have quite good scalability for industrial production. Due to these superior features and their feasibility for solution process, scalability, and surface functionality, various applications of Ti_3_C_2_T_x_, such as in energy storage [56,57,58,59], flexible electronics and biosensors [60,61], adsorption [61,62,63,64,65,66], electromagnetic shielding [67], and anticorrosion [68], have been reported. Therefore, in the review, Ti_3_C_2_T_x_ is taken as a typical representative of the MXene family, and its corrosion-resistant properties for organic composite coatings are discussed.

**Figure 2:**
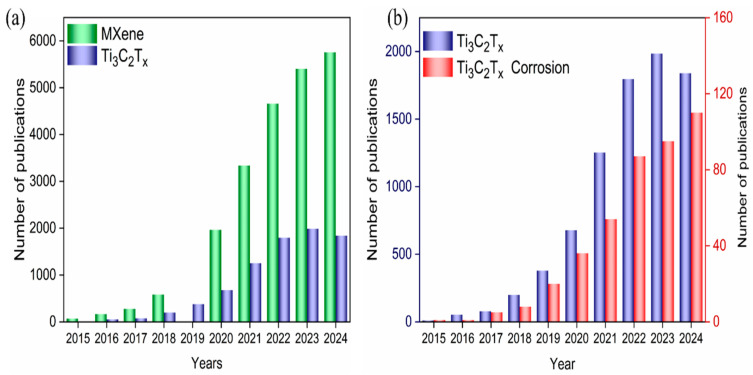
Annual publication of papers on MXene, Ti_3_C_2_T_x_, or “Ti_3_C_2_T_x_ and Corrosion” as a topic over the past decade: (**a**) MXene and Ti_3_C_2_T_x_, (**b**) Ti_3_C_2_T_x_ and “Ti_3_C_2_T_x_ and Corrosion”. Source: Web of Science Search index: [topic = MXene or Ti_3_C_2_T_x_ or “Ti_3_C_2_T_x_ and Corrosion”].

Four groups of topics, namely “MXene”, “Ti_3_C_2_T_x_”, “Ti_3_C_2_T_x_ and Corrosion”, and “Ti_3_C_2_T_x_ and Anticorrosion/Anticorrosive” were selected, and the total and annual number of published papers in the past decade (from 2015 to November 2024, the date of this article) were queried through Web of Science. The results are shown in Figure 2. The annual publication of papers on “MXene”, “Ti_3_C_2_T_x_”, “Ti_3_C_2_T_x_”, and “Ti_3_C_2_T_x_ and Corrosion” is shown in Figure 2a and Figure 2b, respectively. They show that the number of papers on these three topics has grown year by year, indicating that research on MXene and its anticorrosion is receiving increased attention. By November 2024, a total of approximately 417 papers have been published on the topic of “Ti_3_C_2_T_x_ and Corrosion”, with 95 and 110 papers published in 2023 and 2024, respectively. There are 79, 27, and 20 papers, respectively, on the topic of “Ti_3_C_2_T_x_ and Anticorrosion/Anticorrosion”, indicating that research on MXene corrosion and anticorrosion is in the early stage of rapid growth and it is, therefore, worth paying attention.

## 3. Recent Progress of Ti_3_C_2_T_x_-Based Polymer Composite Anticorrosion Coatings

To exploit the corrosion resistance of MXene nanosheets (NSs), the most popular way to prepare the anticorrosion coating is the physical mixing (such as magnetic stirring) of Ti_3_C_2_T_x_ with waterborne epoxy (WEP) or waterborne polyurethane (WPU). In order to prevent agglomeration, various surface-treatment methods have been adopted to improve the dispersibility, compatibility, stability, and orientation properties of MXene. Thus, the discussion in the section is organized as follows: (i) pristine MXenes/polymer matrix composites, (ii) surface functionalization grafting methods, (iii) orientation arrangement, (iv) heterostructure and hybrid composite, and (v) some comparison and summary of the corrosion performance.

### 3.1. Pristine Ti_3_C_2_T_x_

**Figure 3:**
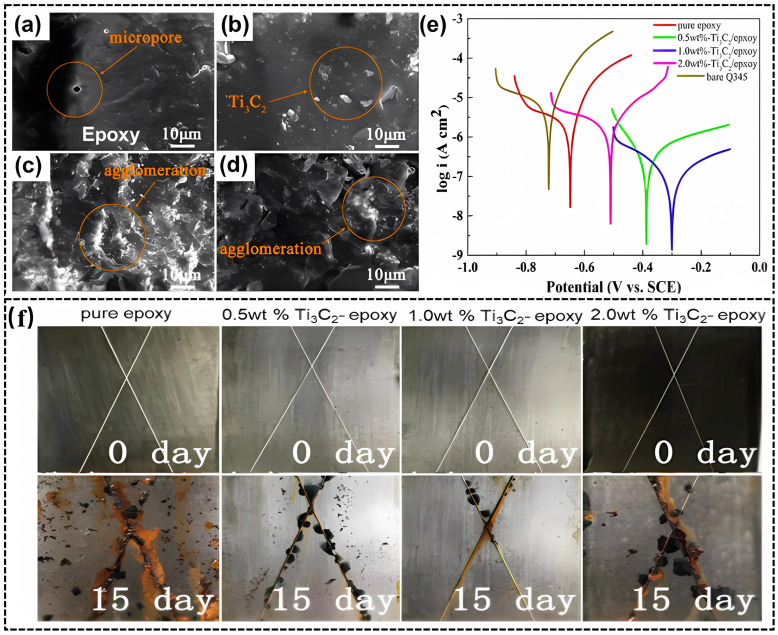
SEM images of surface morphology of MXene/epoxy coating with different Ti_3_C_2_T_x_ content: (**a**) bare EP, (**b**) 0.5 wt.% Ti_3_C_2_T_x_/EP, (**c**) 1.0 wt.% Ti_3_C_2_T_x_/EP, (**d**) 2.0 wt.% Ti_3_C_2_T_x_/EP; (**e**) the polarization curves of uncoated and coated Ti_3_C_2_T_x_/EP samples after 96 h of immersion in 3.5% NaCl, (**f**) photographs of four kinds of coating samples before and after the 15-day salt spray test [30]. Reprinted with permission from [30], copyright 2019, Elsevier.

The first work about the anticorrosive properties of pristine Ti_3_C_2_T_x_ was made in 2019 by Yan et al. [30], in which few-layer Ti_3_C_2_T_x_ NSs were incorporated into EP with an amine curing agent. The effect of Ti_3_C_2_T_x_ content on the morphology, internal structure, and corrosion resistance of the Ti_3_C_2_T_x_/EP composite coating was studied. It is reported that the addition of MXene can prevent the micropores in the cured EP coating from becoming coated on carbon steel Q345 (Figure 3a–d). The Tafel plots (Figure 3e) of four coating samples after 96 h immersion in 3.5% NaCl solution show that the 1.0 wt.% Ti_3_C_2_T_x_ containing composite coating has the highest corrosion potential (*E_corr_*) and the lowest corrosion current density (*I_corr_*), which is 3.39 × 10^−8^ A·cm^−2^, in contrast to that of the pure EP counterpart (1.00 × 10^−6^ A·cm^−2^). As illustrated in Figure 3f, the results of the salt spray test after 15 days indicate that 1.0 wt.% Ti_3_C_2_T_x_ offers the strongest protection. The improved anticorrosion performance is attributed to the barrier effect of MXene flakes as a thin film to inhibit the diffusion of electrolytes, thus providing corrosion protection to the substrate. However, excessive MXenes (such as 2.0 wt.%) will agglomerate in the EP, reducing the anticorrosion performance of composite coatings.

In addition to EP, silane has also been used as a polymer matrix. Polymer composites of Ti_3_C_2_T_x_ and sulfhydryl silane were prepared on copper substrate by Cao et al. in 2022 [69]. The optimal loading amount of Ti_3_C_2_T_x_ is 0.25 Mg/mL. Compared with bare silane coating, the impedance modulus at the lowest frequency (*|Z|*_0.01Hz_) of the silane composite coating is enhanced by 1.8 orders of magnitude, and the *I_corr_* decreases by about 5 times [69].

Besides organic coating, MXene can be adopted to reinforce the corrosion protection of metal matrix composite and metallic coating. The Ti_3_C_2_T_x_/Cu [70] metallic matrix composite and the Ti_3_C_2_T_x_–Ce [71] and Ni–W–Ti_3_C_2_T_x_ [72] composite coating with enhanced corrosion resistance were reported.

### 3.2. Surface Functionalization of Ti_3_C_2_T_x_ MXene

Although MXene has the potential to enlarge the anticorrosion performance of coatings, its enhancement efficiency is lower than expected. The reason for this is that, similar to other 2D materials, MXene also exhibits a strong irreversible stacking and aggregation trend due to interlayer van der Waals forces (vdW) and hydrogen-bonding interactions. Aggregated MXene NSs cannot provide a sufficient protective network and, in the worst case, may affect the continuity of the coating and exacerbate corrosion. In addition, the weak interaction and compatibility between MXene and the coating matrix limit the loading capacity. Therefore, good dispersibility, high compatibility, and moderate content are the primary requirements for preparing high-performance MXene-based composite coatings. Dispersion and compatibility are key to breaking off the MXene conductive network and achieving its maximum blocking effect. Therefore, surface modification of MXene is required to improve its dispersion and compatibility.

Surfactant modification, covalent functionalization, and tuning of surface-functional groups are three effective surface-treatment methods for a nanofiller to disperse homogenously into a polymer matrix [27,29]. The surface terminations, i.e., surface-functional groups (typically –OH, =O, –F, –Cl) existing on the surface of MXene render MXenes hydrophilic. Therefore, it is relatively easy to obtain stable MXene-containing suspensions in water or polar solvents and composites of MXene and polymer due to these groups allowing the reactions and interactions with hydrophilic polymers such as polyacrylic acid (PAA), polydiallyldimethylammonium chloride (PDDA), polyvinyl alcohol (PVA), and many biopolymers. The first MXene polymer nanocomposites (NCs) were reported in 2014 when Ti_3_C_2_T_x_ was introduced to hydrophilic PDDA and PVA polymers [15].

Since this first report, there has been increased interest in the research of MXene polymer NCs. In 2015, Chen et al. prepared one kind of polymer brush structure by grafting poly(2-(dimethylamino) ethyl methacrylate) (PDMAEMA) on vanadium carbide (V_2_CT_x_) via the self-initiated photo-grafting and photo-polymerization of DMAEMA [16]. This NC presented dual CO_2_ and temperature response, showing promise as a stimulus-responsive smart hybrid material that could be applied in biological sensing. In 2017, four functional groups, –NH_2_, –COOR, –C_6_H_6_, and –C_12_H_26_, were grafted onto the surface of Ti_3_C_2_T_x_ by Hao et al. [17] through the introduction of either (3-aminopropyl)-triethoxy-silane (APTES), (γ-methacryl-oxypropyl)-trimethoxysilane (MAPTES), (methyl aniline)-triethoxy-silane (AMTES), or (dodecyl)-triethoxy-silane (DTES). In 2019, Si et al. prepared polystyrene (PS) NCs with colloidal Ti_3_C_2_T_x_, respectively, modified by three types of cationic surfactants [18], namely decyltrimethylammonium bromide (DTAB), octadecyl trimethylammonium bromide (OTAB), and didodecyldimethylammonium bromide (DDAB). It is observed that the basal spacing of Ti_3_C_2_T_x_ increases with the length of the cationic surfactant.

To date, most work has focused on the use of Ti_3_C_2_T_x_. In addition, multiple polymer hosts have been reported, including hydrophilic polymers such as PVA, PAA, silicones, and epoxies.

Functionalizing MXene through grafting modification with coupling agents is one of the most convenient methods to prevent its aggregation and stabilize within the polymer matrix. Certain functional groups grafting onto the active oxidation sites on the surface of MXene will form covalent bonds. Many functional groups can be used to achieve chemical covalent modification, such as organic small molecules, organic polymers, inorganic nano oxides, etc. The most commonly used organic small molecules are various coupling agents, such as silane and titanate.

#### 3.2.1. Functionalization Through Silane-Based Coupling Agents

Owing to the rich reactive surface groups, silane coupling agents have been widely adopted as grafting agents to improve interfacial binding. Surface silanization reaction is beneficial to inhibit its structural degradation, thus effectively stabilizing the MXene and enhancing the surface properties with adjustable hydrophilicity [73,74,75,76,77,78,79,80,81,82,83].

Aminopropyltriethoxysilane (APTES) is the most used silane coupling agent for surface functionalization. The primary amino functional groups of APTES provide a few functionalization feasibilities from bio-conjugation to nanoparticle impregnation. Ji et al. reported that APTES-functionalized Ti_3_C_2_T_x_ demonstrates improved anti-oxidation stability and regulable hydrophilicity [73].

**Figure 4:**
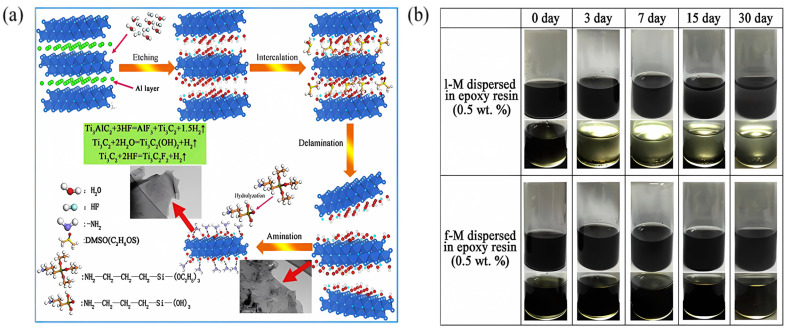
Preparation and stability of amino-functionalized Ti_3_C_2_T_x_ [74]. (**a**) Schematic illustration of synthesis and surface APTES-functionalized Ti_3_C_2_T_x_, (**b**) settlement of 0.5 wt.% l-M and f-M dispersed in EP after 0–30 days of static placement. Reprinted with permission from [74], copyright 2020 Elsevier.

The first work to investigate the corrosion resistance of amino-modified Ti_3_C_2_T_x_ MXene was conducted by Yan et al. in 2020 [74]. The synthesis process of Ti_3_C_2_T_x_ and its surface functionalization through APTES is illustrated in Figure 4a. The photographs of the 0.5 wt.% l-M (pristine Ti_3_C_2_T_x_) and f-M (APTES-modified Ti_3_C_2_T_x_) samples dispersed in EP after 0–30 days are displayed in Figure 4b, where the f-M film remains stable and evenly distributed in the EP slurry for 30 days. After soaking in a 3.5 wt.% NaCl solution for 4 weeks, the f-M 0.5 wt.% composite coating on Al alloy has the highest *|Z|*_0.01Hz_ value of (1.02 × 10^7^ Ω·cm^2^), which was two orders of magnitude higher than that of the bare EP coating. The significant improvement in corrosion resistance is mainly attributed to two points: (a) the good dispersibility and compatibility of f-M flakes in EP, and (b) the interaction between the amino groups of APTES and the hydroxyl groups on the metal surface, which improves the adhesion between the coating and the metal substrate. The work indicates the importance of the ligand functionalization of Ti_3_C_2_T_x_ in enhancing its dispersibility, keeping its chemical stability, and slowing down degradation owing to oxidation in the polymer matrix [74,75,76,77].

Similarly, [3-(2-aminoethyl) aminopropyl]-trimethoxysilane (AEAPTES) was employed to surface-functionalize Ti_3_C_2_T_x_ by Zhang et al. in 2021 [75]. The AEAPTES-modified Ti_3_C_2_T_x_ (named Ti_3_C_2_@Si) was then added into WPU with Ti_3_C_2_T_x_ ratios of 0.05, 0.1, and 0.15 wt.%. The introduction of amino functional groups on Ti_3_C_2_T_x_ facilitates intercalation with isocyanate groups in WPU, causing a strong and compact structure and stable dispersions of Ti_3_C_2_@Si into WPU. Therefore, the formed network of an effective barrier creates complex diffusion paths, slowing the diffusion rates of a corrodent [75]. Importantly, the functionalized Ti_3_C_2_@Si enhanced the hydrophobicity of WPU, which decreased the absorption of water and increased the corrosion performance of Ti_3_C_2_/WPU composite coatings. These findings are similar to those of Gr-based materials, where covalently functionalized Gr showed better corrosion protection than pristine Gr.

In addition to aminosilane, epoxy silane coupling agents were also attempted. In 2022, Li et al. prepared epoxy-functionalized multilayer Ti_3_C_2_T_x_ nanosheets (GPS-Ti_3_C_2_T_x_) by grafting (3-glycidoxypropyl) trimethoxysilane (GPTMS) [78]. They found that the scratch resistance, adhesion, and anticorrosion of the composite coating were all improved, but the flexibility was reduced. The optimal content of GPS-Ti_3_C_2_T_x_ was 0.5 wt.% [78], and the impedance of the corresponding organic coating was nearly three and one orders of magnitude higher than that of bare EP and unmodified Ti_3_C_2_T_x_ coatings, respectively. This article suggests that the performance of GPS-Ti_3_C_2_T_x_ functionalized with epoxy groups should be better than that of APTES-Ti_3_C_2_T_x_ functionalized with amino groups. However, Pourhashem et al. [79] found that amino-functionalized APTES-GO has better dispersion and anticorrosion than epoxy-functionalized GPS-GO, indicating that the modification effects of the same type of silane on MXene and Gr are different.

In 2022, Wang et al. realized silane-grafted Ti_3_C_2_T_x_ by APTES [80]. Due to the chemical reaction between the hydroxyl groups on the MXene and silane molecules, some black dots were observed on the sheets, thus decreasing the transparency of the modified MXene NSs coating. After a 120-day immersion test, the coating resistance was 1.4755 × 10^6^ Ω·cm^2^ [80].

The silane@fresh MXene/WEP coating obtained by Zhou et al. in 2020 demonstrated a high *|Z|*_0.01Hz_ value of 5.5 × 10^8^ Ω·cm^2^ after 40 days’ immersion test [81]. Meanwhile, it kept a larger coating resistance than the oxidized MXene/EP coating after a 60-day immersion test [81].

**Figure 5:**
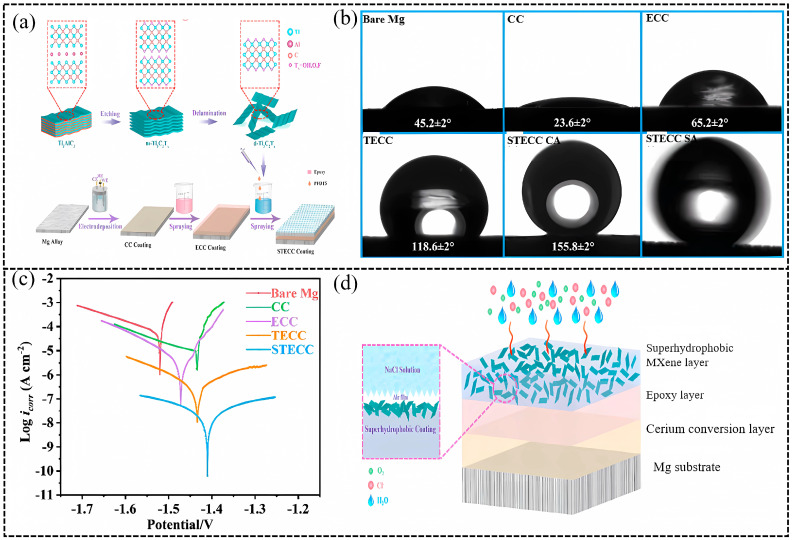
Preparation and performance of functionalized superhydrophobic Ti_3_C_2_T_x_/epoxy/cerium conversion (STECC) composite coating [82]. (**a**) Fabrication process of STECC composite coating on the surface of Mg alloy; (**b**) The WCA of the bare Mg alloy, CC, ECC, TECC, STECC coating, and SA of the STECC coating; (**c**) The polarization curves of as-fabricated specimens after 1 day of immersion in 3.5 wt.% NaCl solution; (**d**) Schematic diagram of the STECC coating corrosion-protection mechanism. Reprinted with permission from [82], copyright 2023, John Wiley and Sons.

In 2023, Yin et al. fabricated a novel superhydrophobic Ti_3_C_2_T_x_/epoxy/cerium conversion composite (STECC) coating with a sandwich structure (Figure 5a) [82]. The water-contact angle (WCA) of the composite coating was 155.8 ± 2° after the fluorosilane modification (Figure 5b). The *|Z|*_0.01Hz_ of the STECC coating was up to 7.49 × 10^6^ Ω·cm^2^, which was larger by four orders of magnitude than the bare Mg alloy (Figure 5c). The corrosion-protection mechanism of the STECC coating is shown in Figure 5d. The superhydrophobic Ti_3_C_2_T_x_ coating on the top avoids the penetration of corrosive mediums and prevents their direct contact with the substrate, solving the problem that the conductive Ti_3_C_2_T_x_ tends to bring out galvanic coupling corrosion. The middle EP coating acts as a corrosion mitigation of the substrate. Furthermore, the cerium conversion coating at the bottom not only increases adhesion between the intermediate layer and the Mg alloy but also forms a passivation layer to protect the substrate. This work provides an effective new strategy for MXene-based composite anticorrosion coatings.

Additionally, MXene NSs-enhanced vinyl ester resin (VER) coatings modified by 3-methacryloxypropyl-trimethoxysilane (γ-MPS@MXene) were developed by Chen et al. in 2023 [83]. The composite coating achieved a remarkable anticorrosion. The 0.02 wt.% γ-MPS@MXene modified coating exhibited significantly lower *I_corr_* than the unmodified counterpart coatings. Furthermore, it shows that the 0.08 wt.% γ-MPS@MXene is the most effective at hindering corrosive media infiltration and safeguarding the carbon steel substrate [83].

#### 3.2.2. Functionalization Through Polymer Molecule

The polymeric layer can be used for surface modification to improve the stability of the MXene [84,85,86,87,88,89,90,91]. Conductive polymers (CPs) such as polythiophene (PT), polypyrrole (PPy), and polyaniline (PANI) exhibit anticorrosion properties. Among them, PANI has received widespread attention for anticorrosion owing to its ease of synthesis, good thermal stability, and reversible acid/base doping/dedoping. As early as 2016, Jafari et al. used PANI-modified Gr as a corrosion-protection layer for Cu and found that the obtained coating had low electrical conductivity and excellent barrier properties, which could prevent water and oxygen molecules from reaching the coating/metal interface [84]. Similar to Gr, the polymerization treatment of MXene is also expected to enhance its corrosion resistance.

**Figure 6:**
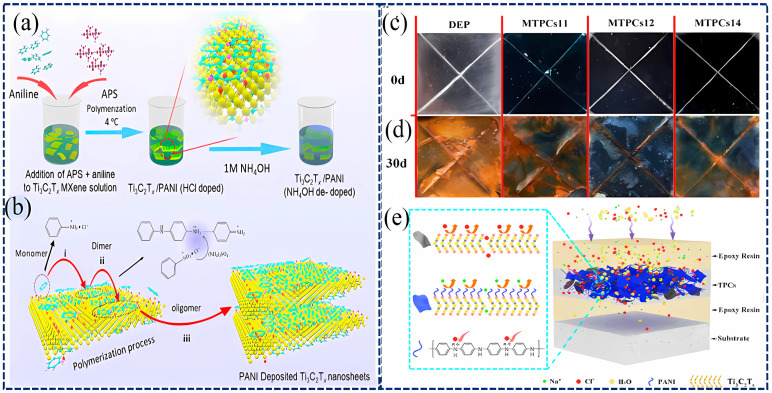
Schematic illustration of the preparation of Ti_3_C_2_/PANI composites [86]. (**a**) Preparation of Ti_3_C_2_ NSs. (**b**) Synthesis of Ti_3_C_2_/PANI composites (TPCs) with the mechanism of oxidative polymerization of aniline on Ti_3_C_2_. (**c**,**d**) Photograph of the samples before and after the salt spray test for 30 days. (**e**) Diagram explaining the corrosion-resistant mechanism of multilayer thin polymer coatings (MTPCs). Reprinted with permission from [86], copyright 2021, Elsevier.

In 2021, Cai et al. studied the interfacial interaction between Ti_3_C_2_T_x_ and Fe and found that the high conductivity of Ti_3_C_2_T_x_ promotes an oxygen-reduction reaction, accelerating the corrosion of low-carbon steel Q345 [85]. Therefore, it is necessary to perform polymerization treatment on MXene to reduce its conductivity. In the same year, they first applied Ti_3_C_2_T_x_@PANI composite materials (TPCs) for the corrosion protection of low-carbon steel [86]. As shown in Figure 6a,b, the multilayer TPCs (MTPCs) with epoxy at the bottom and top and TPCs in the middle were prepared by layer-by-layer (LBL) deposition. By adjusting the PANI content, the conductivity of the composite coating was reduced from 1307 S·cm^−1^ (pristine Ti_3_C_2_T_x_) to 0.5 S·cm^−1^ (TPCs). The photographs of the samples before and after the 30-day salt spray test are displayed in Figure 6c,d, which indicates that the MTPCs12 coating (Ti_3_C_2_T_x_ and aniline mass ratio of 1:2) has the best anticorrosion performance. The corresponding anticorrosion mechanism is illustrated in Figure 6e.

In 2022, Li et al. synthesized a Ti_3_C_2_@PANI composite through in situ intercalation polymerization [87]. It was found that, owing to the synergistic effect of Ti_3_C_2_ NSs’ barrier effect and PANI’s passivation effect, the *|Z|*_0.01Hz_ and charge-transfer resistance of 0.3 wt.% Ti_3_C_2_@PANI/EP coating in 3.5 wt.% NaCl is increased by 1–2 orders of magnitude compared to the bare EP and Ti_3_C_2_/EP. Specifically, the author proposes that Ti_3_C_2_ treated with LiF and HCl accelerates the failure of EP coatings due to the insertion of Li^+^, which results in higher conductivity than the MXene material obtained by HF etching [87].

In 2022, Kaewsaneha et al. explored the corrosion resistance of Zn–EP coatings boosted by Ti_3_C_2_T_x_@PANI NSs hybrid [88]. It shows that, compared with standard EP and Zn–EP coatings, the 2.0 wt.% Ti_3_C_2_T_x_@PANI significantly decreased corrosion products, water absorption, and iron leakage. The superior anticorrosion capabilities are ascribed to the fact that the PANI/@Ti_3_C_2_T_x_ hybrid provided a more effective barrier against corrosion and enhanced the electrical connectivity for reliable cathodic protection.

In 2023, the PANI@Ti_3_C_2_ MXene/polyester-epoxy coating fabricated by Nazarlou et al. [89] exhibited a lower *I_corr_* (9.15 × 10^−7^ A·cm^−2^) than that without PANI-modified coating (2.05 × 10^−5^ A·cm^−2^), and the *|Z|*_0.01Hz_ value increased from ~4 × 10^6^ Ω·cm^2^ to ~8.5 × 10^7^ Ω·cm^2^ [89].

The aforementioned works indicate that the Ti_3_C_2_@PANI composite with lower conductivity can achieve efficient corrosion protection in the EP coating on Q235 steel.

In addition to PANI, chitosan (CS) and polydimethylsiloxane (PDMS) have also been tried [90,91]. In 2022, He et al. successfully grafted MXene with phosphoric acid-modified chitosan (P-CS) due to the strong bond (Ti–O–P) formed through the chemical reaction between the hydroxyl groups of MXene and P-CS [90]. The functionalized Ti_3_C_2_T_x_ MXene that was used as a composite nanofiller for WEP not only improved the dispersion state and compatibility of MXene in WEP but also effectively inhibited galvanic corrosion. After a 50-day immersion test, the *|Z|*_0.01Hz_ of CS@MXene/EP coating was 4.73 × 10^7^ Ω·cm^2^, 4 times larger than that of the MXene/EP coating (~10^7^ Ω·cm^2^) [90].

In 2023, Zhang et al. explored the PDMS-modified Ti_3_C_2_T_x_ MXene to improve the dispersion and stability [91]. Through a silylation reaction, the hydroxyl-terminated PDMS was covalently grafted on the Ti_3_C_2_T_x_ NSs to form the Ti–O–Si bond. The agglomeration of Ti_3_C_2_T_x_ NSs was prevented by the spatial site resistance effect. The *I_corr_* reduces from 3.93 × 10^−7^ (MXene/PU) to 1.49 × 10^−9^ A·cm^−2^ (PDMS@MXene/PU), while the coating resistance increases from 3.02 × 10^4^ to 1.06 × 10^7^ Ω·cm^2^.

#### 3.2.3. Functionalization Through Amine-Based Molecule

With richly hydrophilic groups, polydopamine (PDA) and p-phenylenediamine (PPD) were used to modify the Ti_3_C_2_T_x_ MXene [92,93,94,95,96,97]. Chemical bonds formed between DA and MXene will cause the self-polymerization of DA, which leads to DAs becoming a benzoquinone structure and the MXene NSs being wrapped by PDAs. The PDA molecule in the outer layer of MXene can avoid the further oxidation of MXenes. The interaction between the existing –OH of MXene NSs and the benzoquinone structure of DAs improved the stability of MXene [94]. The PDA@MXene/EP coating kept a higher *|Z|*_0.01Hz_ value at ~10^8^ Ω·cm^2^ than the MXene/EP coating after 30 days’ immersion test [94]. It was found that, compared with the pristine MXene, the DA modification did not damage the 2D structure of MXenes, and the modified MXene nanosheet became thicker [95]. After a 25-day immersion test, the DA-modified MXene/EP coating exhibited a high coating resistance of 6.78 × 10^7^ Ω·cm^2^, and its *|Z|*_0.01Hz_ value (7.94 × 10^7^ Ω·cm^2^) was higher than that of unmodified MXene/EP coating (2.75 × 10^5^ Ω·cm^2^) after a 30-day immersion test [95]. The *I_corr_* value of the modified MXene coating (6.452 × 10^−8^ A·cm^−2^) was lower than that of the unmodified MXene coating (7.004 × 10^−7^ A·cm^−2^). In another work, it shows that after the modification of PDA, the coating resistance and charge-transfer resistance increased from 4250 to 6857 Ω·cm^2^ and from 4.674 × 10^4^ to 2.383 × 10^5^ Ω·cm^2^, respectively [93].

**Figure 7:**
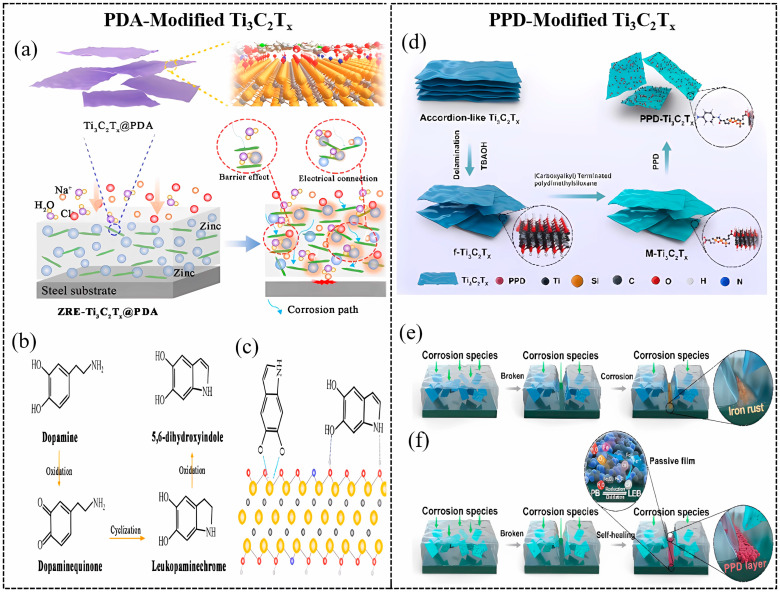
(**a**) The schematic anticorrosion mechanisms of ZRE-Ti_3_C_2_T_x_ @PDA/WEP coating; the mechanisms of (**b**) polymerization of dopamine and (**c**) binding on Ti_3_C_2_T_x_ NSs [96]. Reprinted with permission from [96], copyright 2023 Elsevier. (**d**) The synthesis process for PPD@Ti_3_C_2_T_x_ hybrids. Schematic representation of anticorrosion and self-healing protection mechanism of (**e**) 0.5f-Ti_3_C_2_T_x_/EP, (**f**) 0.5PPD@Ti_3_C_2_T_x_/EP [97]. Reprinted with permission from [97], copyright 2023 Elsevier.

In 2023, Cai et al. reported that PDA-modified Ti_3_C_2_T_x_ significantly enhanced the protective properties of zinc-rich epoxy (ZRE) coating, which contributes to the superior physical barrier and electrical contact effect of ZRE-Ti_3_C_2_T_x_@PDA [96]. After immersion, the corrosion of Zn particles in ZRE resulted in electrical disconnection between Zn particles. By bringing into Ti_3_C_2_T_x_@PDA, the electrical contact between Zn particles and substrate was significantly improved, thereby increasing the cathodic protection of ZRE under long-term immersion. Therefore, due to the physical barrier and conductive properties of Ti_3_C_2_T_x_, effective cathodic protection can be achieved for a longer period of time. The schematic anticorrosion mechanisms of ZRE-Ti_3_C_2_T_x_ @PDA/WEP coating and the polymerization mechanisms of DAs and binding on Ti_3_C_2_T_x_ NSs are displayed in Figure 7a, Figure 7b, and Figure 7c, respectively.

As well as PDA, p-phenylenediamine (PPD) was utilized to functionalize Ti_3_C_2_T_x_ (PPD@Ti_3_C_2_T_x_) via the hydrothermal method by Shi et al. in 2023 [97]. The synthesis process for PPD@Ti_3_C_2_T_x_ hybrids is shown in Figure 7d. The PPD@Ti_3_C_2_T_x_ nanohybrid was adopted to strengthen the EP matrix. Under a 20 MPa alternating hydrostatic pressure (AHP) environment, the *|Z|*_0.01Hz_ of the composite coating enhanced from 3.72 × 10^7^ to 3.74 × 10^8^ Ω·cm^2^ with immersion time increasing from 2 to 6 days. Furthermore, the composite coating presented superior self-healing and great physical barrier behaviors. The anticorrosion and self-healing protection mechanism of 0.5f-Ti_3_C_2_T_x_/EP and 0.5PPD@Ti_3_C_2_T_x_/EP are schematically illustrated in Figure 7e and Figure 7f, respectively.

#### 3.2.4. Functionalization Through Other Organic Compounds

Other compounds have also been explored to functionalize the MXene NSs to enhance their anti-oxidation properties [98,99,100,101,102]. It is known that, despite its superior intrinsic properties, Ti_3_C_2_T_x_ flakes are prone to hydrolysis and oxidation in hydrated environments, causing them to potentially transform into TiO_x_ and TiO_2_. The degradation is a multistep process, usually initiated at the defect and edge sites of MXenes, and the defect sites undergo systematic hydrolysis with TiO_2_ as the outcome of the final formed degraded product. This chemical degradation may impede the anticorrosion behavior of MXene in the long term. Therefore, strategies to mitigate oxidation are requisite.

**Figure 8:**
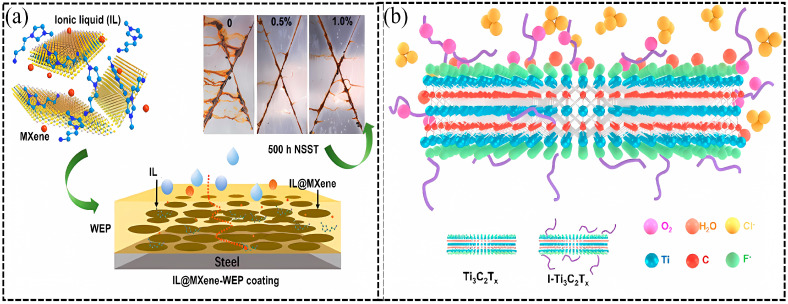
(**a**) Schematic illustration of IL@MXene nanosheets structure, corrosion mechanism of the neat WEP, the IL@MXene/WEP composite coating, and digital photographs of steels protected by different coatings after NSST for 500 h [98]. Reprinted with permission from [98], copyright 2021, American Chemical Society. (**b**) Schematic representation of the corrosion process with I-Ti_3_C_2_T_x_ contained in epoxy resin [99]. Reprinted with permission from [99], copyright 2023 Elsevier.

Recently, it has been discovered that Ti_3_C_2_T_x_ can be passivated by imidazolium salt ionic liquids (ILs), which can quench reactive oxygen species. In 2021, Zhao et al. developed a stable MXene through the noncovalent modification of 1-(3-Aminopropyl)-3-methylimidazolium bromide (APMImBr) on Ti_3_C_2_T_x_ MXene [98]. The tight adhesion of IL loaded on the Ti_3_C_2_T_x_ surface via noncovalent interaction endows the EP composite coating with a good barrier and self-healing properties caused by IL passive films. The IL @MXene NSs structure, corrosion mechanism of the neat WEP, the IL@MXene/WEP composite coating, and digital photographs of different coatings-protected steels after NSST for 500 h are schematically illustrated in Figure 8a.

In 2023, imidazolium salt (1-allyl-3-methylimidazolium iodide) ([AMIm]I) was used to modify the surface of MXene by Ning et al. [99]. The treated Ti_3_C_2_T_x_ was stable for 90 days in an aqueous solution, showing excellent antioxidant stability. The imidazolium ions on Ti_3_C_2_T_x_ MXene inhibit its reaction with O_2_. The adhesion between the coatings and the substrate was significantly enhanced, owing to the strong compatibility between the modified MXene and EP. The anticorrosion behavior of IL-modified Ti_3_C_2_T_x_ is schematically represented in Figure 8b.

Given the increasing awareness of environmental protection, there is an urgent need for environmentally friendly, easily accessible, and corrosion-resistant biological reagents for research on anticorrosion performance, so organic and green modifiers, such as amino acids, are proposed.

In 2021, Li et al. successfully prepared functionalized Ti_3_C_2_T_x_ through a covalent reaction between amino acid (L-cysteine) and Ti_3_C_2_T_x_ surface [101]. Due to the rich groups (–NH_2_, –SH, and –OH) of the L-cysteine, N–Ti and S–Ti bonds will come into being after the condensation reaction between the L-Cys and the MXene. Thus, L-Cys can improve the dispersion and compatibility of MXene in EP and inhibit galvanic corrosion.

Moreover, in 2023, Zhao et al. employed tannic acid (TA) to modify Ti_3_C_2_T_x_ and successfully prepared a parallel arranged P-TA@Ti_3_C_2_T_x_/EP coating via the blade-coating method. The corrosion resistance under alternating hydrostatic pressure (AHP) was investigated [102].

The characteristics of the above-mentioned functionalization grafting methods are compared and summarized in Table 2.

### 3.3. Orientation Regulation

It is known that the incorporation of 2D nanomaterials (NMs) into polymer coatings can significantly boost the physical barrier ability of the composite, as these nanofillers have a sufficiently high aspect ratio to block the diffusion path of a corrosion-related species, forcing corrosion ions to follow a longer, more tortuous path through the coating matrix. However, the anticorrosive performance of 2D NM reinforced-composite coatings usually fails to fulfill the theoretical prediction. This is not only related to the dispersion and compatibility of NMs into the matrix, but more importantly, it is also closely related to their alignment and orientation [2]. In most previous studies, 2D NMs are usually distributed randomly instead of in an orderly mode, which would not achieve optimal barrier performance. The physical barrier of the 2D MXene NSs can be maximized by achieving highly oriented NSs in composite coating. The assembly strategies to obtain high-orientation 2D NM reinforced polymer composite coatings mainly include layer-by-layer (LbL) deposition, magnetic field, electrodeposition, and spin-coating [2].

**Figure 9:**
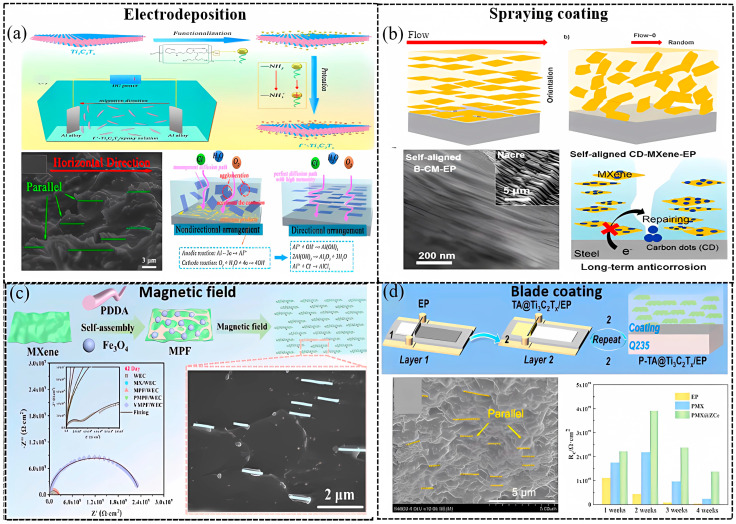
(**a**) Preparation process of orientation aligned Ti_3_C_2_T_x_ MXene/EP coating through electrodeposition [92], including the cross-sectional SEM images and the anticorrosion mechanism. Reprinted with permission from [92], copyright 2022 Elsevier. (**b**) Preparation of oriented CD functionalized Ti_3_C_2_T_x_ MXene via spraying method [104], including the schematic process of airflow-induced self-assembly, the cross-sectional SEM images, and the anticorrosion mechanism of oriented CDs@MXene/EP coating. Reprinted with permission from [104], copyright 2022, Elsevier. (**c**) The MXene/PDDA/Fe_3_O_4_ (MPF) hybrid synthesized via self-assembly induced by magnetic field [107]. Reprinted with permission from [107], copyright 2024, American Chemical Society. (**d**) The preparation process of oriented TA@Ti_3_C_2_T_x_/EP coatings by incorporating tannic acid (TA)-modified MXene into EP using a facile blade-coating method [102]. Reprinted with permission from [102], copyright 2023 Elsevier.

To date, electrodeposition, magnetic field, and self-assembly caused by airflow or blade-coating approaches have been proposed to obtain highly oriented MXenes for anticorrosive coatings [92,102,103,104,105,106,107]. In 2022, Fan et al. obtained well-oriented Ti_3_C_2_T_x_ in EP coating through electrodeposition [103]. The Ti_3_C_2_T_x_ became protonated with a positive charge (f^+^-Ti_3_C_2_T_x_) via amino-functionalization (Figure 9a). The parallel arranged mechanism under the electric field is as follows: the positive f^+^-Ti_3_C_2_T_x_ flakes move towards the cathode under the action of electric field force and rotate in the direction perpendicular to the electric field; meanwhile, the f^+^-Ti_3_C_2_T_x_ flakes with the same charge repel each other, causing the flakes to quickly align parallel to the coating in a short period of time. The EP coating with 1 wt.% f+-Ti_3_C_2_T_x_ exhibits an almost perfect internal aligned structure, and its corrosion resistance is four orders of magnitude higher than that of randomly arranged Ti_3_C_2_T_x_ coatings. After a 4-week immersion test, the *|Z|*_0.01Hz_ of the aligned MXene/EP coating reduced from 6.96 × 10^8^ to 1.05 × 10^8^ Ω·cm^2^, but became 2.63 × 10^4^ Ω·cm^2^ for the randomly distributed coating, while the coating resistances of the aligned and the random MXene/EP coating were, respectively, 1.04 × 10^8^ Ω·cm^2^ and 2.83 × 10^4^ Ω·cm^2^ after the immersion test [92]. The preparation process, the cross-sectional SEM images, and the anticorrosion mechanism of the parallel Ti_3_C_2_T_x_ MXene/EP coating via electrodeposition are displayed in Figure 9a.

In 2022, Ding et al. prepared the carbon dot (CD)-functionalized Ti_3_C_2_T_x_ MXene and realized its alignment in EP coating through an airflow-induced self-assembly method (Figure 9b) [104]. It is found that the oriented distributed NSs can suppress galvanic corrosion, but the random distribution will accelerate the galvanic corrosion on the coating defects. After a 35-day immersion test, the *|Z|*_0.01Hz_ of the oriented CD@ Ti_3_C_2_T_x_/EP coating reduced from 4.1 × 10^10^ to 5.1 × 10^9^ Ω·cm^2^, and that of the random MXene/EP coating decreased from 3.4 × 10^9^ to 1.1 × 10^7^ Ω·cm^2^. The coating resistance of the oriented MXene/EP coating (3.3 × 10^9^ Ω·cm^2^) was higher than that of the randomly distributed one (1.3 × 10^7^ Ω·cm^2^) after the immersion test [104]. The improvement of the stability and durability of the coating is attributed to the elimination of connections of MXene/MXene and MXene/metal perpendicular to the diffusion direction of corrosive media.

In 2024, Fan et al. synthesized an MXene/PDDA/Fe_3_O_4_ (MPF) hybrid via self-assembly induced by a magnetic field (Figure 9c) [107].

Moreover, inspired by the structure of a mille crepe cake, Tan et al. prepared tannic acid (TA)@Ti_3_C_2_T_x_/EP coatings by incorporating TA-modified MXene using a facile blade-coating approach (Figure 9d) in 2023 [102]. The Ti_3_C_2_T_x_ fillers demonstrated near-perfect parallel orientation within the TA@Ti_3_C_2_T_x_/EP composite coating. The *|Z|*_0.01Hz_ of the highly aligned TA@ Ti_3_C_2_T_x_ coating is 1–3 orders of magnitude larger than that of the randomly arranged one. In addition, molecular dynamics (MD) simulation results show that TA molecules can strongly adhere to the substrate in 3.5 wt.% NaCl solution both under a 1 atm and 20 MPa environment, indicating a synergistic protective effect of TA and MXene NSs.

The comparison of the above-discussed methods to obtain highly oriented Ti_3_C_2_T_x_ MXenes NSs in EP composite coatings is listed in Table 3. Each method has advantages and weaknesses. Thus, a strategy to produce highly aligned MXene 2D NSs in composite coatings economically, reliably, and suitable for large-scale preparation is still desired.

### 3.4. Heterostructure Composite

MXenes are prone to agglomeration and oxidation. A heterogeneous structure composite is another strategy to maintain its structural stability and conductivity. Zero-dimensional (nanoparticle (NP), quantum dots (QD)), one-dimensional (nanofiber (NF), nanotube (NT), nanowire (NW)), and two-dimensional (Gr, GO, LDH, h-BN, MoS_2_, etc.) NMs can be adopted to hybridize with two-dimensional MXene to create nanocomposites or heterostructures. By now, carbon-based NMs (typical, CD, CNTs, and Gr and GO) and LDH are two kinds of main NMs used to integrate with MXenes to produce heterostructures [108,109,110,111,112,113,114,115,116,117,118,119,120,121,122,123,124,125,126,127,128].

**Figure 10:**
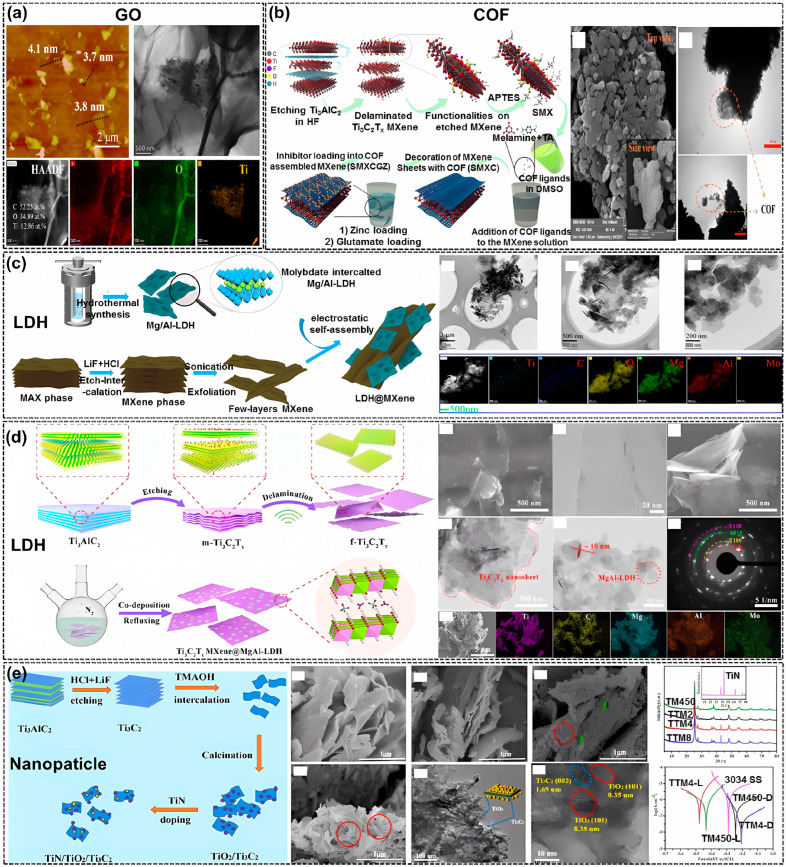
Heterogeneous composite materials formed between multiple NMs and MXenes [4]. Reprinted with permission from [4], copyright 2024 Elsevier. (**a**) AFM and TEM images of GO-Ti_3_C_2_T_x_ NSs, including the HAADF image and EDS mapping of GO-Ti_3_C_2_T_x_ [110]. Reprinted with permission from [110], copyright 2023 Elsevier. (**b**) Schematic illustration of the fabrication process of COF-modified MXene, including FE-SEM and TEM images of synthesized MXene [128]. Reprinted with permission from [128], copyright 2023 Elsevier. (**c**) Synthesis process of Ti_3_C_2_T_x_@MgAl-LDH by the hydrothermal method, including the SEM and TEM images and EDS elemental mappings of the Ti3C2Tx@MgAl-LDH [115]. Reprinted with permission from [115], copyright 2023 Elsevier. (**d**) The co-deposition of Ti_3_C_2_T_x_ MXene@MgAl-LDH heterostructure, including SEM, HRTEM images, SAED pattern, and EDS mapping of Ti_3_C_2_T_x_ MXene@MgAl-LDH [114]. Reprinted with permission from [114], copyright 2021 Elsevier. (**e**) Preparation process of MXene modified by the TiN/TiO_2_ nanoparticles, including the SEM and TEM images of the modified MXene nanosheets, XRD patterns, and polarization curve of the samples [121]. Reprinted with permission from [121], copyright 2022 Elsevier.

Herein, this section is arranged based on the hybridization between MXene and the following NMs: (i) Carbon-based NMs, (ii) LDH, (iii) 0D NPs, (iv) 1D NMs (CNT, CNF), and (v) other 2D NMs, such as h-BN, MoS_2_, and covalent organic frameworks (COFs) [128] or MOF. Some typical heterogeneous composites formed between multiple NMs and MXenes are shown in Figure 10 [4].

#### 3.4.1. Hybridization Between MXene and Carbon-Based NMs

Carbon-based NMs (Gr [108], GO [109,110,111], and CNTs [80,111,112]) have been adopted to complexes with MXene. Gr was first integrated with MXene in 2020 by Yan et al. [108]. It is found that Ti_3_C_2_T_x_ was wrapped by Gr. The Ti_3_C_2_T_x_/@Gr/EP composite coating involving hetero-structured Ti_3_C_2_T_x_@Gr into EP demonstrated excellent anticorrosion properties, exhibiting a corrosion-resistance modulus of 2.14 × 10^9^ Ω·cm^2^, much larger than those of bare EP (1.06 × 10^8^ Ω·cm^2^), Ti_3_C_2_T_x_/EP (1.51 × 10^9^ Ω·cm^2^), and Gr-EP (1.53 × 10^9^ Ω·cm^2^). The boosted performance of the Ti_3_C_2_T_x_/@Gr/EP composite was ascribed to three aspects: (i) protective films formed owing to dual hybrid surfaces, (ii) excellent thermal conductivity and lubricant properties of Ti_3_C_2_T_x_ and Gr, and (iii) the synergistic effects of Ti_3_C_2_T_x_@Gr-interweaved structures that greatly enhanced the corrosion-resistant performance of organic coatings.

In 2021, Shen et al. synthesized GO@Ti_3_C_2_T_x_ heterojunction with the conductivity of 1380 ± 100 S·cm^−1^ and incorporated this nanohybrid into zinc-rich coatings (ZRC) [109]. The ZRC loaded with 0.5 wt.% GO@Ti_3_C_2_T_x_ displayed the highest coating-resistance value (3.047 × 10^4^ Ω·cm^2^) after a 50-day immersion test in 3.5 wt.% NaCl solution, which was one order of magnitude higher than that of other samples.

GO-modified Ti_3_C_2_T_x_ flakes were also prepared by Qiang et al. in 2023 [110]. It shows that the hybridized GO@Ti_3_C_2_T_x_ flake is ~3.9 nm thick and C, O, and Ti elements are distributed uniformly. The *|Z|*_0.01Hz_ of GO@Ti_3_C_2_T_x_/EP coating retained about 1.84 × 10^8^ Ω·cm^2^ after a 8-day immersion test [110].

In 2022, the CDs were used for the surface modification of Ti_3_C_2_T_x_ MXene for corrosion protection by Ding et al. [104]. After a 35-day immersion test, the |Z|_0.01Hz_ of the CDs@Ti_3_C_2_T_x_/EP coating decreased from 4.1 × 10^10^ to 5.1 × 10^9^ Ω·cm^2^ [104].

**Figure 11:**
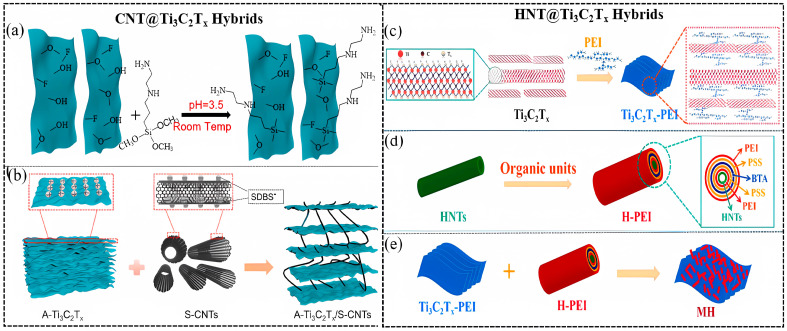
(**a**,**b**) Silanized MXene/carbon nanotube (A-Ti_3_C_2_T_x_/S-CNT) composite structure [80]. (**a**) Schematic diagram of the reaction between Ti_3_C_2_T_x_ flakes and AEAPTMS. (**b**) Schematic electrostatic attraction between A-Ti_3_C_2_T_x_ and S-CNTs. Reprinted with permission from [80], copyright 2022, American Chemical Society. (**c**–**e**) Schematic illustration of the fabrication of MXene@HNTs (MH) and PEI composite [113]: (**c**) H-PEI, (**d**) MXene-PEI, and (**e**) MH. Reprinted with permission from [113], copyright 2022, Elsevier.

In 2022, Wang et al. synthesized the A-Ti_3_C_2_T_x_@S-CNTs/WPU composite coating [80], in which the A-Ti_3_C_2_T_x_ (AEAPTMS-modified Ti_3_C_2_T_x_) became positively charged, and S-CNTs (sodium dodecyl-benzene-sulfonate (SDBS)-treated CNTs) became negatively charged. Therefore, they were able to assemble through electrostatic interaction, preventing their self-aggregation. The optimal content of A-Ti_3_C_2_T_x_@S-CNTs in WPU coatings is found to be 0.2 wt.%. The reaction of Ti_3_C_2_T_x_ flakes and AEAPTMS and electrostatic attraction between A-Ti_3_C_2_T_x_ and S-CNTs are displayed in Figure 11a and Figure 11b, respectively.

In addition, halloysite nanotubes (HNTs) were used to functionalize Ti_3_C_2_T_x_ in 2022 by Deng et al. [113]. The synergistic effects of HNTs and Ti_3_C_2_T_x_ improved the corrosion resistance of the composite coating. The fabrication of MXene@HNTs/PEI composite is shown in Figure 11c–e, respectively.

#### 3.4.2. Hybridization Between MXene and LDH

Due to the inherent positively charged surface of LDH and the negatively charged surface of MXene, a heterostructure of MXene/LDH is easy to obtain via electrostatic interaction [68,114,115,116,117,118]. For instance, MgAl-LDH has been composited with MXene through in situ growing on Ti_3_C_2_T_x_ via the hydrothermal method [114] (Figure 10c) or co-deposition [115] (Figure 10d). After the surface modification of LDH, the Ti_3_C_2_T_x_ MXene retained a sheet-like morphology, but its surface became rougher. After a 21-day immersion test in 3.5 wt.% NaCl solution, the *|Z|*_0.01Hz_ of MXene@LDH was 3.9 × 10^6^ Ω·cm^2^, two times higher than that of EP (2.0 × 10^6^ Ω·cm^2^) [114]. The assembly of these two types of NSs solved their inherent problem of restacking, and the composite still exhibited excellent dispersion and good compatibility with EP after 160 days of standing.

**Figure 12:**
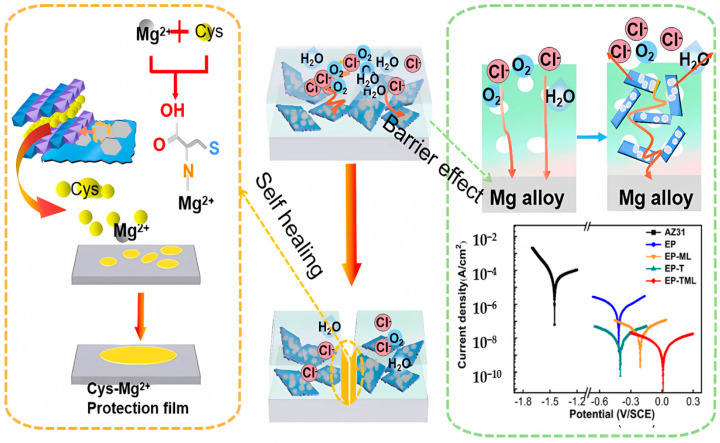
The preparation, corrosion resistance, and mechanism of self-healing of L-Cys-intercalated Ti_3_C_2_T_x_@MgAl-LDH/EP hybrid coating [68]. Reprinted with permission from [68], copyright 2023, Springer Nature.

In 2023, our group prepared a novel Ti_3_C_2_T_x_@LDH/EP hybrid coating using a simple in situ assembled method on AZ31 magnesium alloy [68]. The environmentally friendly organic acid, L-cysteine acid (L-Cys), was encapsulated into MgAl-LDH as a corrosion inhibitor. The Ti_3_C_2_T_x_@MgAl-LDH (TML) heterostructure NSs demonstrate excellent corrosion resistance and self-healing. Its *I_corr_* and *|Z|*_0.01 Hz_ are 1.4 × 10^–9^ A·cm^−2^ and 1.66 × 10^7^ A·cm^−2^, which is 4 orders of magnitude lower than that of the uncoated Mg substrate and 1–2 orders of magnitude larger than that of EP (8.7 × 10^−5^ A·cm^−2^), respectively. The great enhancement of the corrosion-resistant and self-healing performance of the TML/EP composite coating is mainly attributed to the synergistic effect of the “barrier effect” of TML and the corrosion inhibition effect of the L-cysteine acid intercalated in MgAl-LDH. This work proposes valuable insight to widen the functional application of Ti_3_C_2_T_x_ and the design of highly anticorrosive and self-healing organic coatings for Mg alloys. The preparation, corrosion resistance, and mechanism of self-healing of L-Cys-intercalated Ti_3_C_2_T_x_@MgAl-LDH/EP hybrid coating are shown in Figure 12 [68].

#### 3.4.3. 0D NPs/2D MXene Hybrid Structure

Except for the aforementioned carbon-based and LDH NMS, 0D NPs have also been introduced to integrate with 2D MXene [77,104,119,120,121,122,123]. For example, CDs have been used to surface-modify Ti_3_C_2_T_x_ MXene for corrosion protection by Ding et al. in 2022 [104]. The TiN@TiO_2_ NPs were synthesized on the MXene via the calcination and doping process [121]. The formed TiO_2_ NPs were dispersed on both sides of the MXene sheets and between the Ti_3_C_2_ MXene layers. The calcined MXene was observed to possess a rougher surface, a heterostructure of TiN@TiO_2_, and an improved anticorrosion property of the coating.

**Figure 13:**
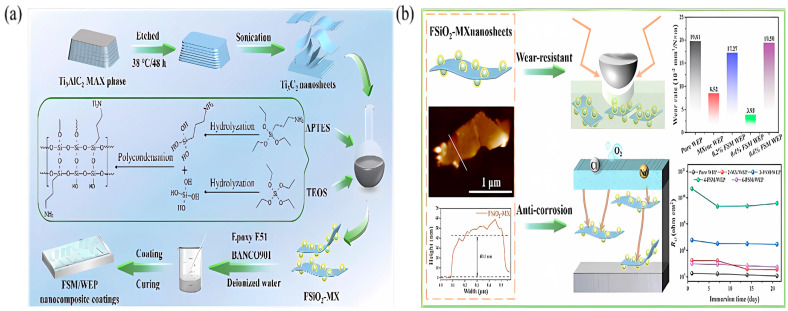
The 0D/2D hybrid structure of in situ growth of aminated silica on MXene nanosheets: (**a**) The fabrication process; (**b**) the structure and antiwear and anticorrosion performance in waterborne epoxy composite coatings [122]. Reprinted with permission from [122], copyright 2022, Elsevier.

In 2022, the aminated silica grown in situ on Ti_3_C_2_T_x_ MXene NSs was adopted as the functional filler by He et al. [122]. The *|Z|*_0.01Hz_ of SiO_2_@ Ti_3_C_2_T_x_/WEP coating was 1.33 × 10^9^ Ω·cm^2^. After a 21-day immersion test in 3.5 wt.% NaCl, the coating resistance was 4.456 × 10^8^ Ω·cm^2^ [122]. The fabrication process, structure, and antiwear and anticorrosion performance of aminated SiO_2_@Ti_3_C_2_T_x_/WEP composite coating are given in Figure 13.

#### 3.4.4. 1D Nanofiber/2D MXene Hybrid Structure

**Figure 14:**
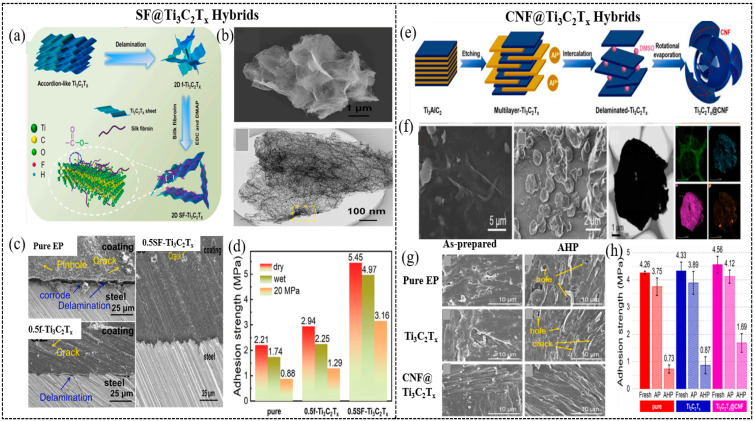
(**a**–**d**) SF@Ti_3_C_2_T_x_/EP hybrid structure [124]: (**a**) Schematic illustration of the preparation procedure of the SF-Ti_3_C_2_T_x_; (**b**) SEM and TEM image of SF-Ti_3_C_2_T_x_; (**c**) SEM images of coating/steel interface for pure EP, 0.5f-Ti_3_C_2_T_x_/EP, and 0.5SF-Ti_3_C_2_T_x_/EP after a 240-h immersion under 20 MPa hydrostatic pressure; (**d**) the adhesion strength for different coatings under dry, wet, and 20 MPa environments. Reprinted with permission from [124], copyright 2021, Elsevier. (**e**–**h**) CNF@Ti_3_C_2_T_x_/EP hybrid structure [125]: (**e**) Schematic representation of the synthesis procedure of the delaminated Ti_3_C_2_T_x_ and CNF@Ti_3_C_2_T_x_ via a wet etching method; (**f**) SEM images of surface and the cross-sectional of Ti_3_C_2_T_x_@CNF film, TEM image of Ti_3_C_2_T_x_@CNF topological structure and its elemental mapping; (**g**) cross-sectional SEM images of bare EP, Ti_3_C_2_T_x_/EP, and Ti_3_C_2_T_x_@CNF/EP coatings before and after under AHP environment; (**h**) adhesion strength of three coatings under freshly as-prepared AP and AHP environments. Reprinted with permission from [125], copyright 2022, Elsevier.

Silk fiber (SF) and cellulose nanofiber (CNF) are two typical cases of 1D nanofiber to complex with MXene [124,125]. In 2021, Chen et al. synthesized a novel silk fibroin-Ti_3_C_2_T_x_ (SF-Ti_3_C_2_T_x_) composite filler [124]. The preparation procedure of the SF-Ti_3_C_2_T_x_ is schematic illustrated in Figure 14a. After SF fiber modification, the surface of MXene NSs became rougher and covered by network materials (Figure 14b), enhancing the adhesive strength between Ti_3_C_2_T_x_ NSs and EP, which is beneficial for obtaining composites with higher density and better mechanical properties. The anticorrosion performance of SF-Ti_3_C_2_T_x_/EP composite coating under atmospheric pressure (AP) and a simulated deep-sea environment was studied, and it was found that the SF-Ti_3_C_2_T_x_-augmented organic coating also has excellent corrosion resistance under high hydrostatic pressure. As shown in Figure 14c, after soaking the 0.5SF-Ti_3_C_2_T_x_/EP composite coating under 20 MPa hydrostatic pressure for 240 h, the interface between the composite coating and steel substrate was tightly integral, without delamination and still had high adhesion strength (3.16 MPa), much larger than that of bare EP (only 0.88 MPa) (Figure 14d).

Furthermore, Wu et al. also designed an MXene-reinforced EP coating (Figure 14e–h) using cellulose nanofiber (CNF)-modified Ti_3_C_2_T_x_ in 2022 [125]. As shown in SEM images (Figure 14g), before immersion in AHP, the three coatings, namely bare EP, Ti_3_C_2_T_x_/EP, and Ti_3_C_2_T_x_@CNF/EP, all presented smooth and intact morphologies without visible microdefects. However, numerous micropores were observed on bare EP coating after a 10-day immersion test under the AHP environment, but the Ti_3_C_2_T_x_@CNF/EP still retained a compact morphology (Figure 14g). Figure 14h shows that the Ti_3_C_2_T_x_@CNF/EP coating has the greatest adhesion strength, regardless of as-prepared, standard AP, or AHP conditions.

#### 3.4.5. Other 2D NMs@2D MXene Hybrid Structure

Other 2D lamellar NMs, such as BN, MoS_2_, MOF, and COF, have also been explored [126,127,128,129,130]. In 2022, Zhou et al. prepared Ti_3_C_2_T_x_-modified h-BN NMs [126]. They show that the Ti_3_C_2_T_x_@BN/EP coating could maintain a time constant after 21 days of soaking in a 3.5 wt.% NaCl solution and its *|Z|*_0.01Hz_ value was 1.5 orders of magnitude higher than that of the bare EP coating.

In 2022, Cai et al. fabricated a Ti_3_C_2_T_x_@MoS_2_ layered hybrid structure through a simple one-step hydrothermal method [127]. The MoS_2_ NSs vertically anchored on Ti_3_C_2_T_x_ NSs took the shape of a unique layered 2D structure that effectively inhibits the restacking of Ti_3_C_2_T_x_ and MoS_2_. The 0.1 wt.% Ti_3_C_2_T_x_@MoS_2_/EP composite coating exhibits the best corrosion-resistant performance.

The COF-modified MXene NSs were synthesized through the in situ growth method by Najmi et al. in 2023 [128]. Owing to the high chemical stability of COFs with imine linkages, the COF-treated Ti_3_C_2_T_x_ MXene presented a stable anticorrosion. After the incorporation of COFs@MXene in the EP coating, the *I_corr_* reduced from 6.701 to 0.815 μA·cm^−2^ [128].

The characteristics of hybridization methods to obtain 0D-,1D-2D NMs/MXene heterostructure are summarized in Table 4.

### 3.5. Comparison of Corrosion Performance of Ti_3_C_2_T_x_-Based Organic Composite Coatings

The comparison of preparation conditions and corrosion resistance of MXene-based organic composite coatings is listed in Table 5. It can be seen that:

(1) The most popular method to prepare anticorrosion coatings is through physical mixing of MXene with polymer. Layered Ti_3_C_2_T_x_ was employed as a filler to disperse in an organic matrix, among which WEP or WPU are the two most commonly used substrates. The loaded amount of Ti_3_C_2_T_x_ in EP-based composite is generally below 2 wt.%, with major cases being 0.5 wt.%. The content of Ti_3_C_2_T_x_ added in WPU-based composite is usually less than 0.2 wt.%, lower than that of WEP-based composite coating.
(2) The anticorrosion performance of organic coatings expressed by the impedance modulus (*|Z|*_0.01Hz_) is approximately on the order of 10^8^ Ω·cm^2^. Among them, the CD-Ti_3_C_2_T_x_/EP coated on Q235 steel has the highest *|Z|*_0.01Hz_ (4.1 × 10^11^ Ω·cm^2^) [104], while that of the APTES-modified A-Ti_3_C_2_T_x_-Ce^3+^/high solid phase EP coated on mild steel is the lowest (4.8 × 10^4^ Ω·cm^2^) [77].
(3) To increase its dispersibility, compatibility, and stability, the Ti_3_C_2_T_x_ MXene is usually required to undergo surface treatment prior to being compounded into a polymer matrix. Surface treatment mainly includes functionalized grafting, surface modification, or hybridization composite with other 2D NMs.
(4) Currently, carbon steel and aluminum alloys are the two most commonly employed metal substrates for MXene-based organic anticorrosive coatings. Only a few studies have been carried out on magnesium [68], and there has been almost no work on titanium alloys, which provides a lot of research space.

**Figure 15:**
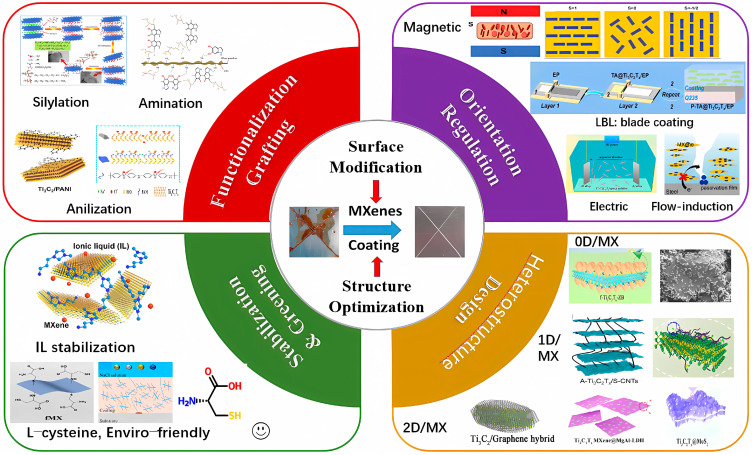
Summary of four surface-modification strategies to improve anticorrosion performance of MXene-based polymer composite coating.

In summary, in order to achieve a high anticorrosion performance of MXene-based polymer composite coating, four surface-modification strategies—surface functionalization grafting, orientation arrangement, heterostructure composite, and antioxidant stabilization and environmentally friendly modification (greening)—were discussed and analyzed. The basic principles and typical cases of four surface-modification strategies are summarized in Figure 15, and the advantages and limitations of these surface-modification methods are given in Table 6.

However, it should be noted that in practical applications, in addition to corrosion-protection performance, other properties, such as self-healing, anti-wear, and weather resistance, are also required. A single protective coating is difficult to meet both the requirements of antiwear and anticorrosion applications in harsh environments [131,132,133]. Therefore, multi-functional composite coatings are needed. To obtain MXene-based organic coatings with self-healing function, the groups of H. Yan (2021) [76], S.A. Haddadi (2021) [77], Z.H. Wang (2023) [68], X. Li (2023) [134], X. Huang (2024) [135], and other researchers have studied the self-healing performance of EP coatings by functionalizing Ti_3_C_2_T_x_ with amino groups and loading-corrosion inhibitors. A few months ago, the self-healing performance and mechanisms of MXene-based coatings for corrosion protection on metals were comprehensively reviewed by Cao’s group [8].

In addition, an inorganic–organic hybridization multilayer protective system composed of MXene is being attempted and deserves attention.

## 4. Conclusions and Prospect

Based on its large specific surface area, abundant surface-functional groups, excellent hydrophilicity, mechanical properties, and ion impermeability, MXene has broad application prospects in the field of anticorrosion. This review focuses on the analysis of organic coatings based on Ti_3_C_2_T_x_ nanostructures. MXene-based composite coating is a holistic system, and its corrosion-resistance behavior mainly depends on the interaction between MXene/polymer and the existing state of MXene in the internal space of the coating. By surface functionalization and composite treatment of MXene, the interface interaction between it and organic substrates can be improved by achieving better dispersion and compatibility. The uniform distribution and good orientation alignment of MXene are important for boosting the shielding effect of MXene on corrosive ions in organic coatings. Methods such as flow induction [104] and electrophoretic deposition [92] can achieve parallel alignment of MXene. Conductive MXene and metal substrates may undergo galvanic corrosion, which can accelerate the local corrosion of the metal. Therefore, it is necessary to reduce the conductivity of MXene, and insulation treatment is one of the feasible solutions.

**Figure 16:**
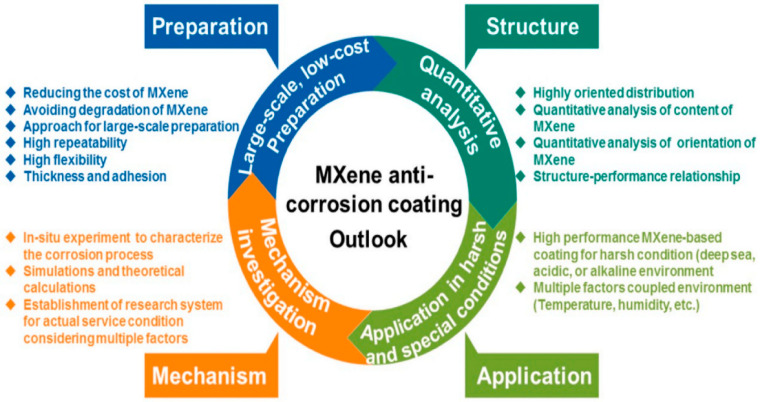
Challenges and opportunities in MXene-based anticorrosion coatings [4]. Reprinted with permission from [4], copyright 2024 Elsevier.

In a critical review in 2024, H. Cao identified the challenges and opportunities to be addressed for MXene-based anticorrosion coatings from four aspects, namely preparation, structure, application and mechanism, and proposed the potential future jobs, which is shown in Figure 16. For more details, please refer to reference [4].

In our opinion, the following main issues still need to be addressed:

(1) The mass production of high-quality, ultra-thin, high-yield, and low-cost single- or few-layer MXenes nanosheets remains a significant challenge. Obtaining customized MXene-based materials with the required wettability, mechanical, chemical, or electrical properties is key to achieving excellent protective performance in applications where the large-scale synthesis of fluorine-free, inexpensive, low conductivity, and highly stable MXene is the foundation. Currently, the exfoliation processes for MXene nanosheets are marked by high costs and low yields, predominantly confined to the laboratory scale. This necessitates the pursuit of cost-effective synthesis methods to make MXene nanosheets more accessible for broader applications.
(2) In terms of the preparation of organic coatings, the uniform dispersion and orientation arrangement of MXene nanosheets are the key factors in determining the anticorrosion performance of composite coatings. Addressing the restacking tendency of these nanosheets, various surface-modification techniques have been employed to prevent their aggregation. However, although covalent modifications are effective, they may disrupt the structural integrity of nanosheets and entail utilizing dangerous chemicals, which pose risks to both the environment and health. Hence, there is a growing call for greener, more sustainable, noncovalent modification methods, such as electrostatic and π–π interactions and hydrogen bonding, which preserve the nanosheet structure while enhancing dispersion and functional integration into coatings. Meanwhile, a simple, efficient, and low-cost coating-processing technology is crucial for industrial applications.
(3) At present, most of the research is conducted on the short-term corrosion behavior of simple components in laboratory environments. To achieve practical applications, it is necessary to conduct in-depth research on the failure mechanism of MXene-based organic anticorrosion coatings in long-term and complex corrosive environments. In addition, the understanding of the mechanism of uniform distribution of MXene and MXene/organic matrix interface is still insufficient, and more advanced experimental tools and/or theoretical methods should be developed to conduct in-depth research to better reveal the relationship between complex interfaces and the corrosion resistance of composite coatings, paving the way to optimize the internal structure of coatings.
(4) At present, the most studied MXene material is Ti_3_C_2_T_x_, so it is necessary to expand the research object to other members of the MXene family. In addition to carbides, it should also be expanded to nitrides and carbonitrides. Meanwhile, apart from Ti-based MXenes, MXenes based on other metals, such as V-, Mo-, W-, etc., are also worth considering. The large family of MXenes will undoubtedly bring more choices for the design of MXene-based anticorrosion coatings.
(5) In practical applications, protection coatings should possess multiple functions. How to apply MXene to obtain composite coatings with synergistic functions such as corrosion resistance, self-healing, self-cleaning, anti-pollution, and antiwear is a new topic worthy of attention. However, to obtain more functionality and higher performance, the difficulty, complexity, and cost of the manufacturing process will undoubtedly increase. Currently, the production process faces hurdles such as high costs, both in the procurement of nanosheets and in the extensive use of organic solvents needed for optimal dispersion, pointing to an urgent need for a balance between synthesis, functionality, cost, and environmental impact.

In short, as a new member of a large class of 2D material, MXene has broad application prospects in the field of anticorrosion. Research at home and abroad is still in its nascent stages, and there is a lot of work worth carrying out. This article aims to provide some useful references and inspirations for researchers engaged in MXene preparation, performance, and MXene-based anticorrosion coatings.

Finally, in order to let readers conveniently understand the many abbreviations that occur in this review, a glossary of acronyms is given below.

## Figures and Tables

**Table 1:** Comparison of the main reviews related to the synthesis and application of the anticorrosive MXene-based polymer composites published after 2022, and the present review.

Title	Focus	Year	Ref.
Ti_3_C_2_T_x_ MXene polymer composites for anticorrosion: an overview and perspective	In this brief review, the current progress of Ti_3_C_2_T_x_ polymer composites for corrosion protection is summarized, and the possible ways to exploit Ti_3_C_2_T_x_ polymer anticorrosive composites more effectively are discussed, and a perspective beyond Ti_3_C_2_T_x_ MXene composition for the development of future anticorrosion coatings is provided.	2022	[3]
Emerging layered materials and their applications in the corrosion protection of metals and alloys	Focus on the emerging 2D materials beyond Gr, TMD such as MoS2, MXenes, LDHs, h-BN, and graphitic carbon nitride in the formulation of effective and protective films and coatings is described and discussed.	2022	[5]
Beyond graphene and boron nitride: why MXene can be used in composite for corrosion protection on metals?	The intrinsic surface protection ability and advantages of MXene beyond Gr and BN for application in corrosion protection are analyzed. The recent progress of experimental results and theoretical calculations are summarized to explore the roles of MXene in anticorrosion. The challenges and perspectives of MXene-based anticorrosion coatings are put forward.	2024	[4]
2D materials for marine corrosion protection: A review	Recent advancements in the development and potential to apply for marine corrosion protection of 2D material (including Gr, LDH, BN, MXenes, and MoS_2_)-enhanced coatings through improved performance metrics and sustainability are reviewed and emphasized. The challenges and further research to overcome application hurdles are highlighted.	2024	[1]
Two-dimensional nanomaterials reinforced organic coatings for marine corrosion protection: state of the art, challenges, and future prospectives	The preparation strategies, properties of 2D NMs, and diverse protection models based on composite coatings for marine corrosion protection, including a physical barrier, self-healing, and cathodic protection, are illustrated. Furthermore, computational simulations and critical factors on the anticorrosion of composite coatings are clarified. Finally, the remaining challenges and prospects for marine corrosion protection based on 2D NMs reinforced organic coatings are highlighted.	2024	[2]
Research progress on MXene-based organic composite anticorrosion coatings	The current research status, mainly before 2023, and challenges and prospects of anticorrosion coatings based on MXene Ti_3_C_2_T_x_ MXene-based epoxy resin organic composite are reviewed and discussed in Chinese.	2024	[6]
Research on two-dimensional layered materials for metal corrosion protection: Advances and challenges	According to material attributes, 2D layered materials (2DLM) are sorted into natural, conventional and emerging three categories. Their recent progress in anticorrosion applications is reviewed with emphasis. The advantages and limitations of various 2DLM-based anticorrosion coatings are compared, and the challenges for future preparation and application in the field of corrosion protection are outlined.	2024	[7]
Toward self-healing two-dimensional MXene coatings for corrosion protection on metals: design strategies and mechanisms	A comprehensive review of the current findings on the anticorrosive and self-healing of MXene-based coating on metals is presented. The review focuses on the design, properties, and mechanisms of four types of self-healing MXene coatings (synergy with Ce^3+^, LDH, inhibitors, and self-healing polymer).	2025	[8]
Surface-modification strategy towards highly anticorrosive Ti_3_C_2_T_x_ MXene-based polymer composite coating: a mini-review	The latest progress in the anticorrosion performance of Ti_3_C_2_T_x_/EP and Ti_3_C_2_T_x_/WPU polymer composite coatings is reviewed. Four surface-modification strategies (functionalization grafting, orientation arrangement, heterostructure hybrid, and stabilization and greening) to improve the dispersion, compatibility, stability, and anti-aggregation properties of MXene are summarized and emphasized. The challenges and opportunities of anticorrosive MXene-based organic composite coatings are prospected.	2025	This work

**Table 2:** Summary of characteristics of functionalization grafting methods.

Functional Groups	Bonding	Typical Examples	Aim	Mechanism	Ref.
Silane-based coupling agent	Covalent	APTES, AEAPTES, GPTMS, MPS	Surface silanization reaction is used to effectively stabilize the MXene against structural degradation and improve the surface properties with adjustable hydrophilicity.	Ti–O–Si bonds and the primary amino functional group make MXene positively charged.	[73,74,75,76,77,78,79,80,81,82,83]
Polymer molecule	Covalent	PANI, PDMS, P-CS	Wrap a layer of polymer insulation on the surface of MXene to reduce its conductivity and passivate it.	Ti–O–Si or Ti–O–P bonds.	[84,85,86,87,88,89,90,91]
Amine-based	Covalent	PDA, PPD	DA undergoes self-polymerization under the action of MXene’s hydroxyl group, forming an insulating polymer that protects MXene from oxidation.	Interaction between the –OH of MXene NSs and DA’s benzoquinone structure improved the stability of MXene.	[92,97]
Other organic compounds	Noncovalent	IL, such as [APMIm]Br, [APMIm]I	Prevent oxidation and improve stability.	ILs isolate oxygen and inhibit oxidation and degradation of MXene.	[98,99]
Other organic compounds	Covalent	Glycine, L-Cys, TA,	Using organic reagents such as amino acids and increasing environmental friendliness.	N–Ti and S–Ti bonds.	[100,101,102,103]

**Table 3:** Summary of the methods to achieve oriented MXenes NSs in composite coatings.

Methods	Advantages	Disadvantages	Ref.
Electrodeposition	Easy to precisely control the structure and suitable for large-scale production	External force required, expensive, high energy consumption	[92,103,106]
Magnetic field	Easy to precisely control the structure	External force required, expensive	[105]
Spraying	Airflow-induced self-assembly, simple process, low cost	Low repeatability, hard to precisely control the structure	[104]
Blade coating	Low cost, alternate	Low repeatability, hard to precisely control the structure	[102]

**Table 4:** Summary of characteristics of heterostructure hybridization methods.

Hybridization	Major Objective	Typical Instances	Ref.
0D/MXene	Simultaneously improve corrosion resistance and wear resistance	CDs, TiO_2_, SiO_2_, TiO_2_/TiN	[77,104,119,120,121,122,123]
1D/MXene	Improve the adhesion between the coating and the substrate	CNT, HNT, CNF	[124,125]
2D/MXene	Preventing agglomeration, provide nanocontainers for corrosion inhibitors to improve self-healing performance	Gr, GO	[108,110]
		LDH	[68,114,115]
		BN, MoS_2_, etc.	[126,127,128]

**Table 5:** Comparison of corrosion performance of Ti_3_C_2_T_x_-based organic composite coatings.

Metal Substrate	MXene	Polymer Matrix	*C_MX_*/ wt./%	Corrosion Performance				Ref.
				*E_corr_*/ V	*I_corr_*/ 10^−9^A·cm^−2^	*R_corr_*/ mm·year**^−^^1^**	*|Z|*_0.01Hz_/ 10^8^Ω·cm^2^	
Steel/Q345	Ti_3_C_2_T_x_	EP (E44)	1.0	−0.30	33.90	3.9 × 10^−4^	6.23	[30]
Cu	Ti_3_C_2_T_x_	Silane	0.25	−0.39	4.04	--	0.19	[69]
Al alloy/6082	APTES-Ti_3_C_2_T_x_	WEP (H228A)	0.5	0.04	1.01	1.10 × 10^−5^	7.94	[74]
Steel/Q235	AEAPTES-Ti_3_C_2_T_x_	WPU	0.1	−0.05	2.67	3.31 × 10^−5^	0.16	[75]
Sn plate	GPTES-Ti_3_C_2_T_x_	EP (E44)	0.5	--	--	--	0.02	[78]
Mild steel	L-Cysteine-Ti_3_C_2_T_x_	EP (E51)	0.2	−0.22	0.12	7.95 × 10^−6^	12.1	[101]
Steel/Q235	P-CS-Ti_3_C_2_T_x_	EP (E51)	0.2	−0.36	5.44	3.53 × 10^−4^	19.8	[90]
Steel/Q235	Ti_3_C_2_T_x_ and TiO_2_	WPU	0.1	−0.04	2.79	3.25 × 10^−5^	0.825	[119]
Steel/Q235	SF-Ti_3_C_2_T_x_	WEP	0.5	--	--	--	8.30	[124]
Steel/Q235	IL-Ti_3_C_2_T_x_	WEP	0.5	−0.81	14		0.016	[98]
Steel/Q235	CD-Ti_3_C_2_T_x_	WEP	0.5	−0.08	--	--	4100	[104]
Al alloy/6082	Parallelized f^+^-Ti_3_C_2_T_x_	WEP	1	--	--	--	3.78 (1 week)	[92]
Steel/Q345	Ti_3_C_2_T_x_@MoS_2_	WEP	0.1	--	--	--	0.018	[127]
Steel/Q345	Ti_3_C_2_T_x_@MgAl-LDH	WEP (H228A)	1.5	--	--	--	0.15	[114]
Steel/Q235	Ti_3_C_2_T_x_/h-BN	WEP	0.5	--	--	--	1.2	[126]
Steel/Q235	AEAPTES-Ti_3_C_2_T_x_/SDBS-CNTs	WPU	0.2	0.72	0.22	--	31.2	[80]
Steel/Q345	Ti_3_C_2_T_x_/PANI	WEP (H228A)	--	−0.11	--	--	7.00	[86]
Steel/Q235	Ti_3_C_2_T_x_@PANI	EP (WH53)	0.3	−0.58	47	3.63 × 10^−4^	6.7 × 10^−4^	[87]
Al alloy/6082	f-Ti_3_C_2_T_x_-ZB(Ti_3_C_2_T_x_/PEI/ZIF-8/BTA)	WEP (H228A)	0.5	--	--	--	4.37 × 10^−2^	[76]
Mild steel	APTES-Ti_3_C_2_T_x_-Ce^3+^	EP (High solid phase)	2.0	−0.67	3610	4.2 × 10^−2^	4.8 × 10^−4^	[77]
Mg alloy/AZ31	Ti_3_C_2_T_x_@MgAl-LDH	WEP (H228A)	0.16	0.01	1.4	--	0.166 (as prepared) 0.00252 (3 weeks)	[68]

Note: *C_MX_*: Content of MXene composite; *E_corr_:* Corrosion potential; *I_corr_:* Corrosion current density; *R_corr_*: Corrosion rate, *|Z|*_0.01Hz_: Impedance modulus. The corrosion performance was measured by immersing in a 3.5 wt.% NaCl solution without scratches for 30 min. Abbreviation: EP: Epoxy resin; WEP: Waterborne epoxy resin; PU: Polyurethane; WPU: Waterborne polyurethane; APTES: 3-aminopropyltriethoxysilane; AEAPTES: 3-(2-aminoethylamino)-propyl] trimethoxysilane; P-CS: Phosphorylated chitosan functionalized; GPTES: (3-glycidoxypropyl) trimethoxysilane; SF: Silk fibroin protein; IL: Ionic liquid; CD: Carbon quantum dots; SDBS: Sodium dodecylbenzenesulfonate; PANI: Polyaniline; PEI: Polyethyleneimine; ZIF-8: 2-methylimidazole; BTA: Benzotriazole.

**Table 6:** Comparison of advantages and weaknesses of the surface-modification methods to improve corrosion performance of Ti_3_C_2_T_x_-based organic composite coatings.

Modification	Advantage	Disadvantage
Functionalization grafting	Versatile coupling agent; wide range of function adjustment; from non-conductive to conductive, from hydrophilic to hydrophobic	Design at atomic/molecular scale; complex preparation process; cost; reagents may not be environmentally friendly
Orientation regulation	Simple design idea; without new additive	Limited to flat surface; difficulty in uniform parallel arrangement; poor adhesion between coating and substrate
Heterostructure Nanocomposite	Simple design; versatile nanomaterials to choose from; easy-to-obtain multifunctionality (self-healing, etc.)	Complex preparation process; difficult to obtain precise layer-to-layer assembly; cost; unclear interaction between the heterostructure and the coating
Stabilization	Ionic liquids isolate oxygen, prevent oxidation, and improve stability	Limited types of reagents; cost; unclear interaction between the heterostructure and the coating
Greening	Using organic reagents such as amino acids; environmentally friendly	Limited available types of organic reagents; poor adhesion between coating and substrate

## Data Availability

No new data were created or analyzed in this study.

## List of Acronyms

0D	Zero-dimensional
1D	One-dimensional
2D	Two-dimensional
3D	Three-dimensional
AEAPTES	3-(2-amino-ethylamino)-propyl] trimethoxysilane, C_8_H_22_O_3_N_2_Si, KH-792
AHP	Alternating hydrostatic pressure
[AMIm]Br	1-allyl-3-methylimidazolium bromide
[AMIm]I	1-allyl-3-methylimidazolium iodide
AMTES	Anilino-methyl-triethoxy-silane, C_13_H_23_NO_3_Si, KH-ND42
AP	Atmospheric pressure
APTES	3-aminopropyl-triethoxy-silane, C_9_H_23_NO_3_Si, KH-550
[APMIm]Br	1-aminopropyl-3-methylimidazolium bromide
BTA	Benzotriazole
CD	Carbon quantum dots
CNT	Carbon nanotube
COF	Covalent organic frameworks
CP	Conductive polymers
CS	Chitosan
CVD	Chemical vapor deposition
DA	Dopamine, C8H11NO2
DDAB	didodecyldimethylammonium bromide
DTAB	decyltrimethylammonium bromide
DTES	Dodecyltriethoxysilane, C_18_H_40_O_3_Si
E_corr_	Corrosion potential
EIS	Electrochemical impedance spectroscopy
EP	Epoxy resin
GDP	Gross domestic product
GPTES	(3-glycidoxypropyl) trimethoxysilane
GO	Grapheme oxide
Gr	Grapheme
h-BN	Hexagonal boron nitride
HNTs	Halloysite nanotubes
I_corr_	Corrosion current density
IL	Ionic liquid
LbL	Layer-by-layer deposition
L-Cys	L-cysteine amino acid
LDH	Layered double hydroxide
LEIS	Local electrochemical impedance spectroscopy
MAPTES	γ-methacryloxy-propyl-trimethoxysilane, C_10_H_20_O_5_Si, KH-570
MOF	Metal organic framework
MPS	3-methacryloxy-propyltrimethoxy-silane
MTPCs	Multilayer Ti_3_C_2_T_x_/PANI composite
MXene	2D transition metal carbides/nitrides
NF	Nanofiber
NP	Nanoparticle
NMs	Nanomaterials
NSs	Nanosheets
NT	Nanotube
NW	Nanowire
OTAB	Octadecyl trimethylammonium bromide
PAA	Polyacrylic acid
PANI	Polyaniline
PBZ	Polybenzoxazine
PDA	Polydopamine
PDDA	Poly(diallyldimethylammonium chloride)
PDMS	Polydimethylsiloxane
PDMAEMA	Poly2-dimethylaminoethyl metchacrylate
PEI	Polyethyleneimine
PPD	Phenylenediamine
PPy	Polypyrrole
PS	Polystyrene
PT	Polythiophene
PU	Polyurethane
PVA	Polyvinyl alcohol
QD	Quantum dots
SDBS	Sodium dodecylbenzenesulfonate
SF	Silk fiber
STECC	Superhydrophobic Ti_3_C_2_T_x_/epoxy/cerium conversion composite coating
TA	Tannic acid
TML	Ti_3_C_2_T_x_/MgAl-LDH heterostructure
TPCs	Ti_3_C_2_T_x_/PANI composite materials
vdW	van der Waals
VER	Vinyl ester resin
WCA	Water-contact angle
WEP	Waterborne epoxy
WPU	Waterborne polyurethane
|*Z*_0.01Hz_|	Impedance modulus corresponding to 0.01 Hz
ZIF-8	Zeolitic imidazolate framework-8
ZRC	Zinc-rich coatings
ZRE	Zinc-rich epoxy coating
